# Optimized culture of primary human alveolar type II cell–derived 3D organoids from fibrotic lung tissue with phenotypic and metabolic profiling

**DOI:** 10.1186/s12931-026-03610-9

**Published:** 2026-03-07

**Authors:** Lara-Jasmin Schröder, Julia Rückoldt, Stephanie Schubert, Lars Knudsen, Sabina-Marija Janciauskiene, Christopher Werlein, Mareike Knoll, Regina Engelhardt, Christina Petzold-Mügge, Jonas C. Schupp, Marius M. Hoeper, Jens Gottlieb, Fabio Ius, Patrick Zardo, Marc Lindenberg, Christian Riehle, Lavinia Neubert, Jan C. Kamp

**Affiliations:** 1 Institute for Pathology, Hannover Medical School, Hannover, Germany; 2 German Center for Lung Research (DZL), Biomedical Research in End-Stage and Obstructive Lung Disease Hanover (BREATH), Hannover, Germany; 3 Department of Respiratory Medicine and Infectious Diseases, Hannover Medical School, Hannover, Germany; 4 Institute of Functional and Applied Anatomy, Hannover Medical School, Hannover, Germany; 5 Department of Cardiac, Thoracic, Transplant and Vascular Surgery, Hannover Medical School, Hannover, Germany; 6 Institute for Medical Microbiology, Hannover Medical School, Hannover, Germany; 7 Department of Cardiology and Angiology, Hannover Medical School, Hannover, Germany

**Keywords:** Alveolar type II cells, Lung epithelium, Lung organoids, Alveolosphere, Idiopathic pulmonary fibrosis, Human lung explant, Fibrotic end-stage

## Abstract

**Background:**

Alveolar type II (AT-II) epithelial cells are essential for alveolar repair, immune regulation, and surfactant secretion. Despite their promise for pulmonary disease modeling, limited access and culture methods hinder translational use. We established a patient-derived 3D AT-II organoid system from fibrotic and non-fibrotic lung tissue to maintain AT-II-associated features, enable cryopreservation, and capture disease-associated metabolic alterations.

**Methods:**

HT-II-280^+^ AT-II cells were isolated by magnetic bead sorting from 63 lung tissues (15 idiopathic pulmonary fibrosis, 26 secondary fibrosis, 22 tumor-distant controls). Cells were expanded as organoids in 3D culture from initial passage 0 up to passage 3. AT-II-associated features were assessed by immunofluorescence, flow cytometry, and transmission electron microscopy. Cryopreserved cells were recovered after ≥ 28 days and tested for viability and organoid-forming capacity. Metabolic profiling was performed using extracellular flux assays.

**Results:**

AT-II cells were successfully (~ 80%) isolated and combined with a serum- free feeder-free culturing approach to reproducibly generated alveolospheres with highly efficient colony formation (> 90% in P1), especially in AT-II cells from fibrotic explants. Primary tissue-derived lung organoids display heterogeneous morphologies and sizes, most prominently in fibrotic-derived cultures, as indicated by histology and microcomputed tomography. Culture conditions were optimized to minimize differentiation towards AT-I cells or dedifferentiated epithelial states with partial basaloid features. Expression of key AT-II-associated markers (proSP-C, HT-II-280), and the presence of lamellar bodies were maintained across passages at the population level. Cryopreservation maintained high viability, organoid-forming capacity, and metabolic activity, enabling long-term storage. Fibrotic organoids exhibited disease-associated metabolic reprogramming characterized by a pronounced glycolytic shift with increased ATP production.

**Conclusion:**

We established a reproducible cell-line-free 3D culture system from primary human AT-II cells of end-stage ILD lungs to generate patient-derived lung organoids. These organoids maintain AT-II-associated features across passages, remain viable after cryostorage, and capture disease-associated metabolic reprogramming. Fibrotic-derived AT-II cells consistently demonstrated a Warburg-like glycolytic phenotype, reflecting increased energy demand. This scalable model in vitro provides a defined resource for mechanistic studies of epithelial dysfunction in pulmonary diseases and supports biobanking for future precision medicine applications.

**Supplementary Information:**

The online version contains supplementary material available at 10.1186/s12931-026-03610-9.

## Introduction

Idiopathic pulmonary fibrosis (IPF) is a progressive and fatal interstitial lung disease (ILD) characterized by excessive extracellular matrix (ECM) deposition, loss of alveolar structure, and respiratory failure. The disease primarily affects older adults and has a poor prognosis, with a median survival of only 3–5 years after diagnosis [1]. Current anti-fibrotic therapies can slow progression but neither halt nor reverse fibrosis [2]. The lack of curative options highlights the urgent need for human-derived disease models that capture key aspects of IPF pathogenesis [3].

Alveolar type II (AT-II) epithelial cells play a central role in alveolar homeostasis and fibrotic remodeling. These cuboidal surfactant-producing cells constitute ~ 5% of the alveolar surface, secrete surfactant, regulate immune responses, and act as progenitors for alveolar type I (AT-I) cells [4]. Under normal conditions, AT-II cells self-renew and transdifferentiate into AT-I cells after injury [4]. In IPF, however, AT-II cells undergo apoptosis, senescence, or aberrant activation, contributing to chronic epithelial damage and fibrosis [5]. In addition to these processes, AT-II cells in fibrotic lungs can give rise to aberrant epithelial states with basaloid-like features, a transitional phenotype observed in distal fibrotic regions of IPF [6–8]. Recent studies, including single-cell transcriptomic analyses, suggest that matrix stiffening and altered mechanical cues drive this basaloid differentiation program [6]. Such stiffness-induced epithelial transitions have been demonstrated in vitro and in vivo, where direct contact with rigid substrates promotes loss of AT-II-associated features and induction of basal markers [6, 8, 9]. They may further sustain the fibrotic niche by secreting profibrotic cytokines, undergoing epithelial–mesenchymal transition (EMT), and potentially contributing to fibroblast activation [10]. AT-II cells can be identified by lineage-defining markers such as surfactant proteins (SFTPC, SFTPB), ABCA3 [11], LAMP3 [12], NKX2-1/TTF-1, and the surface marker HT-II-280 used for human isolation [13, 14]. Morphologically, the presence of lamellar bodies is a characteristic hallmark of AT-II cells [15, 16]. AT-I cells, in contrast, cover ~ 95% of the alveolar surface and express RAGE/AGER, HT-I-56, and HOPX [17, 18]. In addition to AT-II–derived fibroblast-like states, injured AT-I cells in IPF adopt dysfunctional/senescent programs that secrete pro-fibrotic mediators (e.g., TGF-β, CTGF, WNT ligands), thereby activating fibroblasts and amplifying ECM deposition; AT-I–restricted regulators such as AGER (RAGE) and caveolin-1 further modulate this crosstalk, and their dysregulation has been linked to heightened fibrosis [19–22].

Recent studies showed that IPF-derived AT-II cells undergo metabolic reprogramming from oxidative phosphorylation to aerobic glycolysis, a “Warburg-like” phenotype. This is linked to mitochondrial dysfunction, elevated glycolytic enzyme expression (LDHA, PDK1), lactate accumulation, and suppression of pyruvate dehydrogenase via the PDK1–HIF-1α axis [23–25]. Such energetic shifts are thought to drive fibrotic remodeling and represent potential therapeutic targets.

Organoids—self-organizing 3D epithelial culture systems—have become valuable models for disease research. Pulmonary organoids derived from fetal, adult, or induced pluripotent stem cell (iPSC) sources can recapitulate aspects of alveolar architecture and function [26–28]. They allow long-term expansion, passaging, and cryopreservation facilitating applications in disease modeling, drug testing, and personalized medicine [29, 30]. Several lung organoid models reproduced features of fibrosis, epithelial plasticity, and anti-fibrotic responses [31, 32]. Compared to 2D cultures, organoids better preserve epithelial polarity, surfactant secretion, and progenitor functions. Importantly, 2D monolayer systems are known to induce rapid AT-II to AT-I transition, loss of surfactant production, loss of apical–basal polarity, and unphysiological spreading, making them unsuitable for maintaining key AT-II–associated features of primary human epithelium [4, 27, 33]. By providing a compliant 3D environment and sustained cell–cell interactions, organoid systems more effectively preserve AT-II-associated phenotypic hallmarks such as lamellar body abundance. However, standardized robust primary human AT-II organoid models from end-stage fibrotic lungs are still limited. Most existing models rely on murine or iPSC-derived epithelium and do not reflect the native metabolic or regenerative state of diseased AT-II cells.

Moreover, current systems often lack reproducibility across laboratories and restricted scalability, restricting their broader translational use. A reproducible, feeder-free human AT-II model that allows cryopreservation and biobanking would therefore be a a valuable technical resource for cross-center studies and future precision medicine approaches.

Here, we establish a defined feeder-free 3D culture system for primary human HT-II-280 + AT-II cells isolated from both healthy and fibrotic (IPF and secondary fibrosis) lung tissue adapted from previous work by *Konishi *et al*.* [33] and *Katsura *et al*.* [27]*.* preserve key AT-II–associated features across passages as assessed by canonical markers (HT-II-280, proSP-C, lamellar bodies), (ii) evaluate cryopreservation and the feasibility for organoid biobanking, and (iii) assess metabolic differences between healthy and fibrotic AT-II organoids using extracellular flux analysis. We hypothesized that fibrotic AT-II cells exhibit enhanced glycolytic metabolism compared to controls, reflecting disease-associated metabolic reprogramming. By providing a defined and transferable protocol, our study establishes a reproducible human AT-II organoid culture framework that supports mechanistic studies of epithelial dysfunction in ILD.

## Methods

### Patient cohort and tissue collection

For this study, we analysed 63 lung tissues (22 females, 40 males; average age 60 ± 12 years), including 41 fibrotic explants (15 IPF, 26 secondary pulmonary fibrosis) and 22 tumor-distant “healthy” controls. Tissues were used either for one or multiple investigations. Donors provided written informed consent, and the study was approved by the Hannover Medical School ethics committee (8867_BO_K_2020, 10194_BO_K_2022). Patient demographics and details on donor usage across all assays, including whether individual donors contributed to organoid characterization, immunofluorescence, FACS analysis, or Seahorse extracellular flux experiments, are summarized in Table 1. Furthermore, per each experiment or technique, defined numbers of N from fibrotic or “healthy” from this cohort were utilized as also indicated in corresponding figure legends. Donors used exclusively for metabolic assays are annotated with the letter “K”. Due to the large number of parallel analyses and the requirement for early-passage material, not all donors included in the Seahorse cohort could be used for additional characterization assays.

**Table 1:** Patient cohort. demographics, diagnosis and experimental utilization of material for this study

EXPLANTS				
No	SEX	AGE	DIAGNOSIS	UTILIZATION
1	m	60	IPF	B
2	m	66	IPF	B
3	m	60	IPF	B
4	m	60	IPF	B, D
5	m	68	IPF	C1, H
6	m	56	IPF	C1, F, H
7	m	61	IPF	E, F, K
8	m	45	IPF	E, K
9	m	59	IPF	K
10	m	66	IPF	E, K
11	m	62	IPF	C1, G
12	m	63	IPF	C2, H
13	w	63	IPF (+ EAA)	K, H
14	m	65	IPF	H
15	m	58	IPF	B
16	m	48	sec. Fibrosis (EAA)	C1
17	m	67	sec. Fibrosis (EAA)	C1
18	m	54	sec. Fibrosis (ReTx)	B, D, F, G
19	w	67	sec. Fibrosis (RA rheumatic arthritis)	C2
20	w	59	sec. Fibrosis (RA rheumatic arthritis)	I, J
21	w	66	sec. Fibrosis (EAA)	E
22	w	61	sec. Fibrosis (EAA)	C1
23	m	57	sec. Fibrosis (EAA)	B, F, G, K
24	w	23	sec. Fibrosis (COPA)	A, B, F, G, I, J
25	m	32	sec. Fibrosis (EAA)	A, B, D, E, J F, G
26	m	33	sec. Fibrosis (Lupus erythematodes)	B, D, E, G, J, I, K
27	m	60	sec. Fibrosis (systemic)	B, I, J, K
28	m	58	sec. Fibrosis (Sjögren-Syndrom/COPD)	E
29	w	53	sec. Fibrosis (RA rheumatic arthritis)	E
30	w	55	sec. Fibrosis (Influenza-A associated)	C2
31	m	66	sec. Fibrosis (Lupus erythematodes)	A, B
32	m	64	sec. Fibrosis (RA rheumatic arthritis)	B
33	m	69	sec. Fibrosis (UIP)	B, F
34	m	63	sec. Fibrosis (UIP/RA rheumatic arthritis)	B
35	m	44	sec. Fibrosis (Sars-CoV-2 associated)	B
36	m	65	sec. Fibrosis (EAA)	B
37	m	65	sec. Fibrosis (unclear)	C2
38	w	64	sec. Fibrosis (EAA)	K, L
39	m	48	sec. Fibrosis (CPFE)	K
40	m	64	sec. Fibrosis (amy. Dermatomyositis)	K
41	m	61	sec. Fibrosis (NSIP/Psoriasis)	B

TUMOR-DISTANT CONTROL TISSUE				
No	SEX	AGE	DIAGNOSIS	UTILIZATION
1	m	75	NSCLC	B
2	m	58	Metastasing Rectum Carcinom, Lung Carcinom	B
3	w	67	NSCLC	B, F
4	w	69	NSCLC	C1
5	m	67	NSCLC	B, C1, D, F, G, H, K, L
6	w	57	NSCLS	C1, D, I, J,H
7	w	67	Unclear circular lesion, COPD	B, C1, H, I, J, K
8	m	87	NSCLC	B, C1, H, J, K
9	m	72	NSCLC	B, C1, I, J
10	w	62	Adenocarcinoma	B, C2, F, G
11	w	25	Pleurodesis	A, B, E, I, J
12	w	73	Complete lobectomy	A, B, E
13	w	56	NSCLC/Adenocarcinoma	A, B, E, K
14	w	68	NSCLC, Mamma-Carcinom, COPD	E, K
15	w	59	Adenocarcinoma, Mamma-Carcinom	C2
16	m	66	NSCLC, Mamma-Carcinom	I, J
17	m	69	Unclear; Rectal Cancer	G
18	w	36	Neuroendocrine tumor	B, K
19	w	77	Squamous cell carcinoma/COPD, following chemotherapy	K
20	m	70	Squamous cell carcinoma	G
21	w	57	Mamma-Carcinoma, following chemotherapy	E
22	m	71	Basal cell carcinoma	L

Assay Code

A Cytospin

B Brightfield/Colony Formation Rate

C1 Flow cytometry- organoids(HT-II-280, proSP-C, HT-I-56, Lysotracker, Annexin V)

C2 Flow cytometry- organoids(HT-II-280, proSP-C, HT-I-56, Lysotracker, Annexin V)

D Imaging/TEM

E H&E

F IF- whole mount

G IF- cross-sections

H Live Dye IF(Lysotracker)

I Live Dead IF

J LDH & WST

K Extracellular Flux

L IF- basaloid characterization

### Isolation of primary alveolar epithelial cells

Primary human alveolar type II (AT-II) and type I (AT-I) cells were isolated from freshly explanted fibrotic or tumor-distant “healthy” control lung tissue by magnetic-activated cell sorting (MACS) using the AT-II marker HT-II-280 and the AT-I marker HT-I-56, respectively [13, 17, 27, 33]. All steps were performed on ice or at 4 °C unless otherwise specified and all buffers and consumables were pre-cooled.

Peripheral lung tissue was dissected free of pleura, bronchi, and large vessels, mechanically minced, and enzymatically digested (Miltenyi Biotec, gentleMACS and No. 130–110–201 Multi Tissue Dissociation Kit 1) [Miltenyi Biotec, Cat. No. 130–110–201]). After sequential filtration (100/70 µm), centrifugation (450 g, 7 min, 4 °C), and erythrocyte lysis with ACK lysis buffer (Thermo Fisher Scientific/Gibco, Cat. No. A10492-01) followed by sequential filtration (40 µm), cells were washed and resuspended in MACS buffer (1% BSA, 2 mM EDTA in PBS).

Following Fc receptor blockade, (10 µL/sample; Miltenyi Biotec) for 15 min at 4 °C, cells were incubated with 5 µL mouse IgM anti-HT-II-280 antibody (Terrace Biotech, Cat. No. TB-27AHT2-280; 1:50 in MACS buffer) for 1 h at 4 °C with gentle rotation (60 rpm). For AT-I isolation, the primary antibody was replaced by mouse IgG anti-HT-I-56 (Terrace Biotech, Cat. No. TB-29AHT1-56) at the same dilution.

Cells were washed with 1.5 mL MACS buffer, centrifuged, and resuspended in 2 mL MACS buffer containing anti-mouse IgM or anti-mouse IgG MicroBeads (Miltenyi Biotec; 20 µL beads per 10^7^ cells, diluted 1:10 in MACS buffer). Bead incubation was carried out for 30 min at 4 °C with gentle rotation. After washing, suspensions were applied to pre-equilibrated LS columns in an OctoMACS separator. Negative fractions were collected as flow-through, while positive fractions were eluted after column removal from the magnet. Both fractions were pelleted, resuspended in MACS buffer, and used for counting or downstream applications. MACS was selected over FACS to reduce shear stress and preserve viability for organoid culture.

Both positive and negative fractions were pelleted (450 g, 7 min, 4 °C) and resuspended in 200 µL MACS buffer for counting or immediate downstream applications.

### 3D organoid culture

MACS-isolated HT-II-280^+^ AT-II cells were pelleted (450 g, 7 min, 4 °C) and resuspended in serum-free, feeder-free (SFFF) medium based on Advanced DMEM/F-12 (Gibco, Cat 12,634,028) supplemented with a variety of proliferation enhancers e.g. GSK3β-Inhibitor CHIR99021 and AT-I cell differentiation inhibitors such as ROCK-Inhibitor Y-27632, TGF-β-Receptor I (ALK5)-Inhibitor SB431542 and p38 MAPK-Inhibitor BIRB796 (see Supplementary Table S1). SFFF medium was adapted from *Konishi *et al*.* [33] using the same components but with modified concentrations to optimize maintenance of AT-II cell phenotype and increased proliferation. Cells in SFFF medium were mixed with Corning® Matrigel® Growth Factor Reduced Basement Membrane Matrix (Cat. No. 356231) at a 5:8 ratio (cell suspension:matrigel) and seeded as 100–130 µL domes in 24-well.

#### Technical note

As Matrigel is subject to lot-to-lot variability in composition and mechanical properties, the optimized medium:Matrigel ratio described here may require minor adjustment depending on batch-specific characteristics, which could influence reproducibility across laboratories.

For all experiments, domes were seeded with exactly 60,000 cells independent of donor origin, fibrosis status, or passage number, ensuring that differences in organoid abundance or colony formation reflected biological rather than technical variation. After polymerization (20–30 min, 37 °C), 500 µL SFFF medium was added per well. For the first 4–5 days, IL-1β was added to enhance alveolosphere formation. Culture medium also contained EGF and FGF-10 to support Wnt and ERK/AKT pathway activation, essential for AT-II expansion. Organoids were grown for up to 16 days and passaged up to P3, with medium exchanged every 2–3 days.

To generate single-cell suspensions for passaging of AT-II cells, organoids were segregated using TrypLE™ Select Enzyme (Gibco, Cat. No. 12563011). Per matrigel dome, 500 µL TrypLE™ Select Enzyme were utilized to firstly mechanically disrupt domes and incubate them at 37 °C for 15–30 min with regard to organoid sizes and numbers. Suspensions were again mechanically disrupted, washed 3 × in PBS, and centrifuged at 500 g for 5 min before further processing.

#### Technical note

For all histological investigations and especially for flow cytometry labeling, we strongly recommend the utilization of Cell Recovery Solution (Corning, Cat. No.: 354253) before further processing. 300 µL Cell Recovery Solution were added to the domes to dissolve organoids from matrigel for more surface efficient stainings (e.g. such as for HT-II-280 labeling) and minimization on unlabeled cells in FACS to to residual matrigel. Organoids free of matrigel can further undergo immediate trypsination.

### Cryopreservation

For cryopreservation, organoids were mechanically and enzymatically dissociated as described above. Up to 500,000 single cells were resuspended in 1 ml cryomedium (70% FBS, 20% SFFF medium, 10% DMSO, added dropwise). Cryomedium was incubated with AT-II for 20 min at RT to allow for proper diffusion of DMSO. Cryovials were placed in a Mr. Frosty™ container at –80 °C and transferred after 24 h to –150 °C for long-term storage. To assess safety and viability, samples were stored ≥ 28 days at –150 °C. For recovery, cells were thawed in a 37 °C water bath for 2 min, diluted in 14 ml Advanced DMEM/F-12 at room temperature, incubated for 10 min, centrifuged (7 min, 450 g), and directly reseeded in Matrigel at a 5:8 ratio.

### In-depth characterization of alveolar organoids

To confirm the identity and purity of alveolar organoids over multiple passages and to evaluate cryostorage potential, a multitude of techniques was applied including conventional histology, microcomputed tomography, transmission electron microscopy, image analysis, immunofluorescence & flow cytometry as well as viability assays. For the sake of space, these techniques are described in detail in the supplement.

### Extracellular flux assays

To assess the metabolic properties of fibrotic vs control organoids, glycolytic capacity and ATP production of fibrotic and healthy tissue-derived AT-II cells measured by extracellular flux assays using the *Agilent Seahorse XF HS Mini Analyzer S7852A* platform (Agilent, Santa Clara, CA). Organoids from P0-P1 were separated to single-cell suspension using trypsination as previously described in *method section*. Next, 12,000 cells from P1-P2 were plated 10–12 d prior to experiments in each well of *XFp PDL Miniplates* (Agilent, Cat. No.: 03722–100) in matrigel domes with 50 µl total volume as suggested by *Ludikhuize *et al. [34] and provided with SFFF media. Later passages were intentionally excluded to avoid culture-induced metabolic drift and maintain biological fidelity. By day of experiment, each well therefore roughly contains 85,000–120,000 cells as organoids. Samples were prepared in duplets. Cartridges were prepared corresponding to the manufacturer’s instructions for *Seahorse XFp Glycolysis Stress Test Kit* (Agilent, Cat. No.: 103017–100) and *Seahorse XFp Real-Time ATP Rate Assay Kit* (Agilent, Cat. No.: 103591–100). On the day of experiment, assay media were freshly prepared. SFFF was aspirated from matrigel domes exhibiting normal sized alveolospheres after cultivation miniplates and exchanged with 100 µl assay media 45 min prior to measurement, resulting in a total volume of 150 µl per well. Of note, after serial titration experiments for *Seahorse XFp Glycolysis Stress Test Kit,* we increased the concentrations of glucose to 15 mM, oligomycin to 5 µM and 2-DG to 200 mM per well, each resuspended in assay medium. Each injection port contained 20 µl volume. We measured 3 cycles each basal curve, glycolysis, and glycolytic capacity followed by 5 cycles of glycolytic reserve asessement. Similarly, for *Seahorse XFp Real-Time ATP Rate Assay Kit,* oligomycin (5µM) and Retenone + Antimycin A (2µM) per well were utilized in higher concentration than recommended from Agilent for measurement of 2D cell culture and with a volume of 20 µl per injection port. These concentrations are roughly in line with concentration utilized to measure extracellular flux of intestinal organoids by *Ludikhuize *et al. [34]. ATP production were assessed by 3 cycles each basal ECAR and OCR, inhibition of mitochondrial ATP synthesis, and, finally, mitochondrial-associated acidification. Following measurements, organoids were collected from matrigel using Cell Recovery Solution (Corning, Cat. No.: 354253) and harvested for DNA isolation using QIAamp DNA micro kit (Qiagen, Cat. No.:56304) and samples were measured by Nanodrop to enable a normalization of experimental values relative to approximated number of cells. Additionally, BrdU assays (Cell Signaling Technology, Cat. No.: 6813S) were performed to confirm unaltered cell proliferation and viability post-extracellular flux assays.

### Statistical analysis

**Fig. 1:**
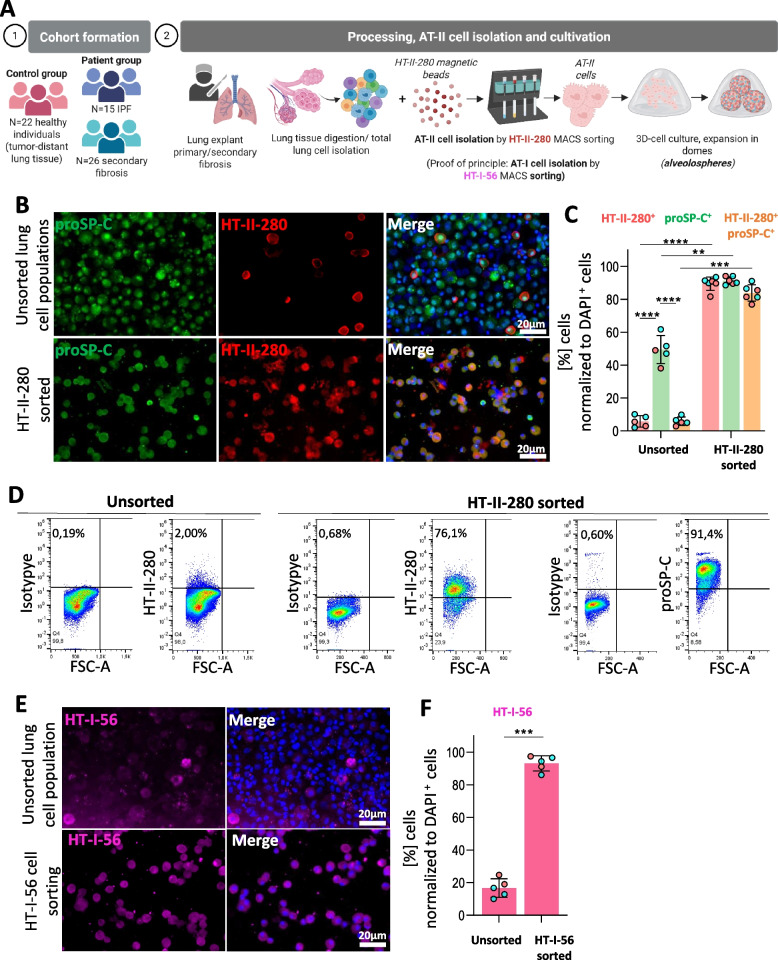
Isolation of AT-II and AT-I epithelial cells by magnetic bead sorting. **A**. Schematics of sorting-based isolation. Cohort of this study included 63 lung tissues derived from *N* = 15 IPF and *N* = 26 secondary pulmonary fibrosis patients in addition to *N* = 22 tumor-distant, pathologically inconspicuous control tissues in total. Per each experiment or technique, defined numbers of N from fibrotic or “healthy” from this cohort were utilized as indicated in corresponding figure legends. Primary AT-II cells were isolated from human lung explants or partial tumor resections. Following magnetic bead sorting by HT-II-280, AT-II cells were cultured in matrigel domes to form alveolospheres. **B**. Exemplary display of cytospins of unsorted whole lung cell population vs. freshly MACS sorted cells using anti-HT-II-280 mouse IgM reveal the expression of AT-II-specific markers. HT-II-280 (red) and proSP-C (green) localize to AT-II cells. Images of unsorted and sorted cells in cytospins are derived from the same patient. Cell nuclei were stained in blue using DAPI staining. Representative picture of 5 patient tissues for sorted fraction (*N* = 2 from healthy tumor-distant control tissue, *N* = 3 from fibrotic end-stage lungs) and of 4 patient tissues for unsorted fraction (*N* = 2 from healthy tumor-distant control tissue, *N* = 2 from fibrotic end-stage lungs). Scale bars = 20 µm. **C**. Quantification of HT-II-280^+^ proSP-C^+^ cells/total to DAPI^+^ cytospinned cells following directly after HT-II-280 MACS indicate an overlapping population of both AT-II cell markers. Individual data points display the mean of 5 images recorded per each patient tissue. One-way ANOVA (***, *p* = 0.0079) shows statistical significance between means of single- or double positive cell populations before and after sorting which is further demonstrated in Tukey ‘s multiple comparison test indicated in graph. Sorted fraction displays insignificant differences (ns, *p* = 0.0974 for HT-II-280^+^ vs. HT-II-280^+^ proSP-C^+^ cells; ns, *p* = 0.0631 for proSP-C^+^ vs. HT-II-280^+^ proSP-C^+^ cells). However, HT-II-280^+^ are strongly enriched after sorting (****, *p* < 0.0001) alongside proSP-C^+^ cells (**, *p* = 0.0091) or double-positive cells (***, *p* = 0.00411). Representative picture of 6 tissues (*N* = 3 from healthy tumor-distant control tissue, *N* = 3 from fibrotic explants). **D**. Exemplary flow cytometry measurement before and directly after MACS-based sorting illustrates strong enrichment of HT-II-280^+^ cells directly post-sorting. This indicates that a relevant fraction of surface epitopes remains accessible despite prior bead binding, although potential epitope competition or steric hindrance should be considered when interpreting absolute signal intensities. Majority of sorted cells further express proSP-C. Gating was performed to exclude debris and focus on single cells, see Supplementary Fig. 1. Representative data of total *N* = 7 (*N* = 3 healthy tissue donors and *N* = 4 fibrotic explants). **E**. Cytospins of unsorted whole lung cell population vs. freshly MACSed cells using anti-HT-I-56 ms IgG highlights the suitability to also isolate AT-I cells from the same tissue with similar approach. Cell nuclei were stained in blue using DAPI staining. Representative picture of 4 tissues (*N* = 2 from healthy tumor-distant control tissue, *N* = 2 from fibrotic explants). Scale bar = 20 µm. **F**. Quantification of HT-I-56 expression in cytospinned cells. Individual data points display the mean of 5 images recorded per each patient tissue. Numbers of HT-I-56-expressing cells relative to DAPI staining. Paired t-test showed significant enrichment of HT-I-56.^+^ following sorting (***, *p* = 0.00452). Representative picture of 5 tissues (*N* = 2 from healthy tumor-distant control tissue, *N* = 3 from fibrotic explants)

**Fig. 2:**
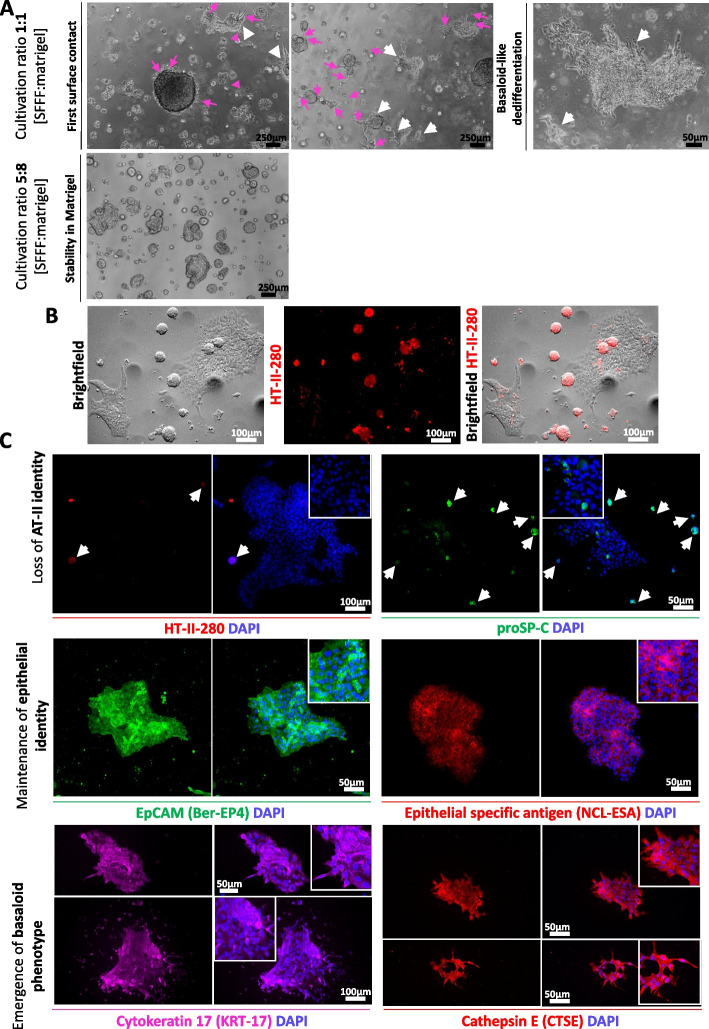
Titration of medium to matrigel ration for 3 d dome culturing. **A **The appropriate ratio for 14 d 3D-culturing of freshly isolated HT-II-280 + cells was determined by matrigel titration experiments. When implementing culturing for human primary AT-II cells from fibrotic explants, we sarted by utilizing 1:1 ratio of matrigel and AT-II cell culture medium SFFF as previously published by *Konishi *et al*.* However, considering domes with larger volume 100–130 µl and corresponding larger surface area, the 1:1 ratio led to sinking of organoids inside the dome and direct contact to plastic well, resulting in the transformation of HT-II-280 + cells to basaloid cells as observed by morphology after 7–10 days of culturing (right panel). Upon initial contact with plastic, visible flaking of AT-II cells occurs on the surface of the existing organoids (pink arrows). After prolonged sinking and contact with rigid plastic, visible spreading and transformation into basaloid morphology occurs (white arrowheads). Therefore, ratio was adjusted to 5:8 Medium: Matrigel for 3D culturing for this study, resulting in proper embedding of cells for prolonged cultivation (left panel). **B** Side-by-side comparison of AT-II organoids and emerging basaloids 7–10 days from 1:1 medium:Matrigel ratio display loss of HT-II-280 expression upon contact to plastic or glass surface. Brightfield image and HT-II-280 expression. Representative image of stiffness-induced dedifferentiation of AT-II cells from fibrotic explant. Scale bar = 50 µm. **C** Confirmation of basaloid cell identity by immunofluorescent staining. To validate transformation into basaloid cells following utilization of 1:1 ratio, cells were cultured for 7–9 days on glass slides and stained to illustrate the loss of AT-II identity by decline of HT-II-280 and proSP-C (upper panel). Here, white arrowheads indicate expression of AT-II markers in remaining small organoids. The maintenance of epithelial identity by EpCAM or Epithelial specific antigen is highlighted the middle panel. The emergence of a basaloid phenotype was demonstrated by expression of Cytokeratin 17 (KRT-17) and Cathepsin E (CTSE; lower panel). For epithelial markers, representative pictures of AT-II cells from *n* = 2 tumor-distant control tissues are shown; AT-II markers as well as basaloid markers were confirmed on *n* = 2 tumor-distant control tissues and *n* = 1.fibrotic explant Scale bars = 50 µm; 100 µm for HT-II-280

**Fig. 3:**
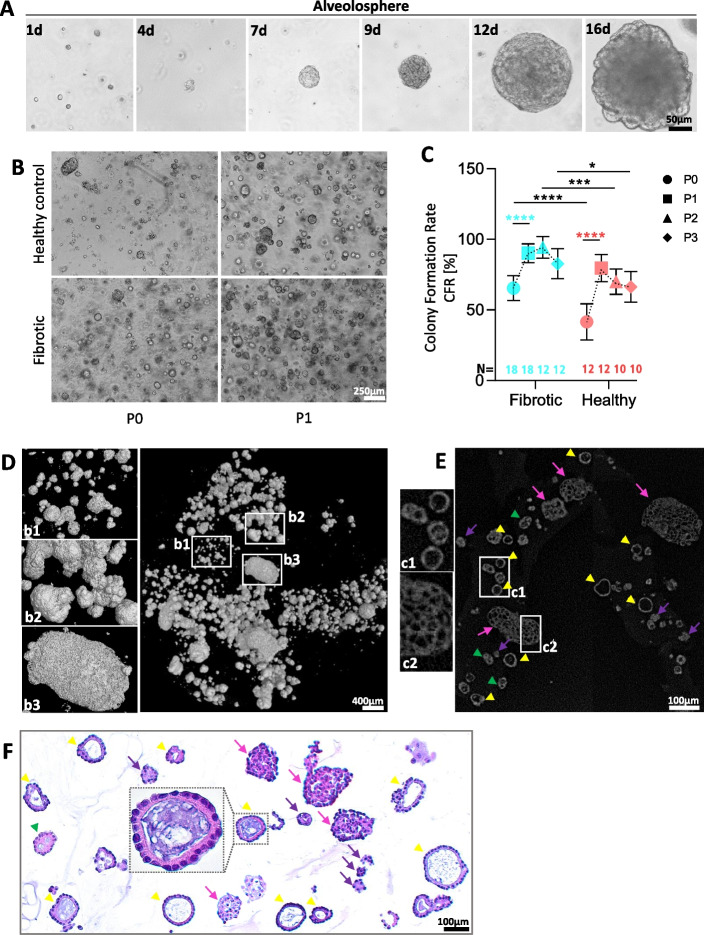
Morphology and growth pattern of primary human AT-II organoids during 3D culturing. **A** Representative bright-field images of an alveolosphere emerging from HT-II-280^+^ cells. Formation of AT-II alveolospheres in 3D matrigel domes cultures over the course of 14 days in vitro following HT-II-280^+^cell population isolation. Scale bars in all images equals 50 µm except last overview of airway organoid (= 100 µm). **B** Expansion of organoid culture at 12 d in P1. Bright-field images, Scale bar = 250 µm.** C** Colony formation rate (CFR) of AT-II cells from initial passage 0 to passage 3. For quantification, for each patient ID, 5 bright field images from B. were counted at 40 × magnification for the relative number of colonies (called alveolospheres) on day 7 of culture. Data points represent mean of *N* = 12–18 fibrotic explant and *N* = 10–12 tumor distant control tissue-derived AT-II organoid cultures per passage. Group analysis, summary data displayed as mean ±. Red = healthy background, blue = fibrotic background. Following two-way ANOVA (****, *p* < 0.0001), Tukey’s multiple comparisons were performed to illustrate the difference in colony formation between fibrotic and healthy tissue-derived AT-II cells each passage (P0: ****, *p* < 0.0001, P1: n; *p* = 0.3197,P2: ***p = 0.0002, P3: *p = 0.0373) as indicated (black asterisks), while blue (fibrotic, ****, *p* < 0.0001) and red asterisks (control, ****, *p* < 0.0001) show significant increase of formed colonies between P0 und P1 for both backgrounds. **D** Exemplary plastic overview of lung organoids in microcomputed tomography derived from a fibrotic explant at 12 days post-isolation (first passage). NanoTom scan images. Representative examples of *N* = 2 organoid cultures from IPF patients and *N* = 2 tumor-distant tissues from resections. Zoom-in windows highlight heterogeneous sizing and variance in shape of organoids per passage. Scale bar = 400 µm. **E** Volume rendering of lung organoids during microcomputed tomography revealed the presence of smaller (50–70 µm diameter) mono- (yellow arrowheads) and polycystic (green arrowheads) alveolospheres with lumen. Other smaller (< 100 µm, purple arrows) and larger organoids (> 150 µm, pink arrows) are completely filled/layered out with AT-II cells and display a honeycomb-structure. Representative examples of *N* = 2 organoid cultures from IPF patients and *N* = 2 tumor-distant tissues from resections. Scale bar = 100 µm. **F** Histopathology of alveolar organoid cultures. Representative image of lung organoid FFPE sections in hematoxylin and eosin (H&E) staining. Organoids from *N* = 7 fibrotic patients and *N* = 3 healthy tissues were investigated. Scale bar = 100 µm. Mono- (yellow arrowheads) and polycystic (green arrowheads) organoids with lumen. Smaller (< 100 µm, purple arrows) and larger organoids (> 100 µm, pink arrows) with lumenless-structure

**Fig. 4:**
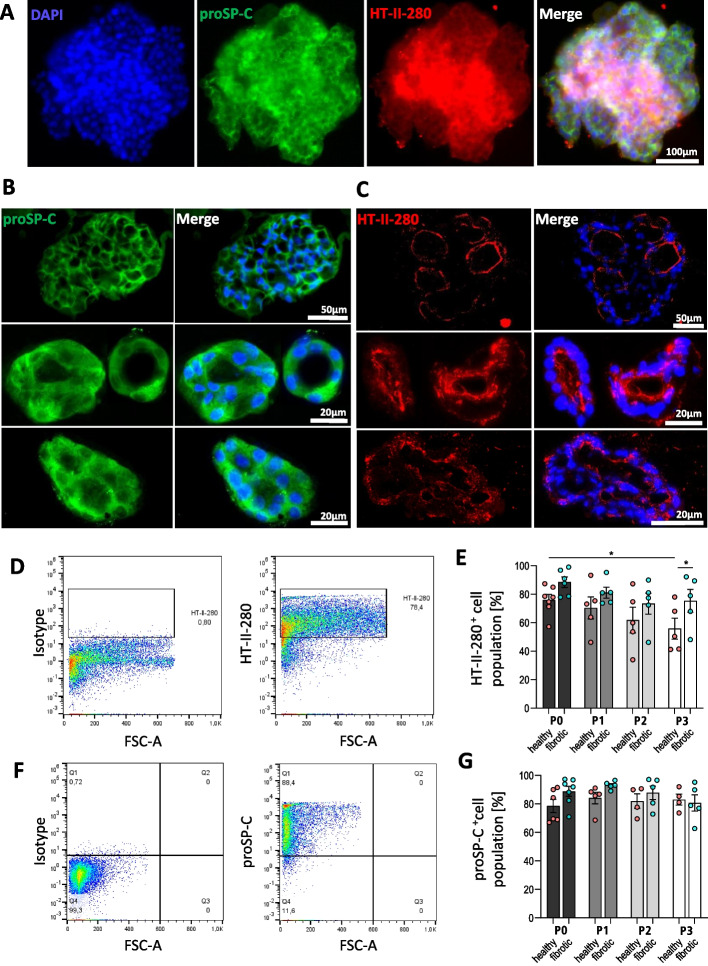
Primary human lung organoids from explants and tumor-distant control tissues display expression of AT-II associatedmarkers after 3D-culturing. **A** Whole mount immunofluorescence labeling illustrates that the surface of lung organoids from AT-II cells respond to HT-II-280 and proSP-C expression (example from P1, 12 d, fibrotic explant). All replicates (*n* = 5 fibrotic *n* = 3 healthy controls from tumor-distant tissues, P0-P2) express proSP-C and HT-II-280 at the surface. Scale bar = 100 µm. **B** Representative images from cryosection immunolabeling of alveolospheres indicate expression of secreted proSP-C throughout the lumen of lung organoids (and) and in the extracellular space. Representative images of proSP-C staining on larger (top panel), and smaller alveolospheres (middle and bottom panel) with diverse morphology. *N* = 7 fibrotic *N* = 4 healthy controls from tumor-distant tissues (P0-P2). Scale bar = 50 µm or 20 µm, respectively. **C** Fluorescent images demonstrate the predominant localization of HT-II-280 in the apical plasma membrane of ATII cells (red) as well as some intracytoplasmic staining and luminal accumulation/apical shedding. Representative images of staining on larger polycystic (top panel), small monocystic (middle panel) or small polycystic (bottom panel) alveolospheres from *N* = 6 fibrotic and *N* = 6 healthy controls from tumor-distant tissues (P0-P2). Scale bar = 50 µm or 20 µm, respectively. **D** Representative flow cytometry measurement for HT-II-280 on human AT-II cells from organoids tracked from initial passage 0 up to passage 3. Primary anti-HT-II-280 ms IgM was conjugated to gt anti-ms IgM Alexa Fluor-488 (displayed in FITC channel). Full gating strategy for P0 is displayed in Supplementary Fig. 2. Example from cells dissociated from alveolospheres at Passage 0, fibrotic explant. **E** Quantification of HT-II-280 expression in cells from lung organoids measured by flow cytometry until passage 3. Statistical analysis showed significant difference in HT-II-280 expression between P0 and P3 (two-way ANOVA *, *p* = 0.0426; Tukey ‘s multiple comparison test *), but no difference between cells derived from fibrotic explants (IPF and secondary fibrosis, turquoise) or healthy tumor-distant lung tissues (red) except in P3 (two-way ANOVA *, *p* = 0.0288; Tukey ‘s multiple comparison test *). Cells derived from *N* = 5–6 fibrotic tissues and *N* = 5–6 tumor-distant control tissues. **F** Representative flow cytometry measurement for proSP-C in human AT-II cells from organoids displayed per passage. Primary anti- proSP-C rb polyclonal was conjugated to gt anti-rb (H + L) Alexa Fluor-488 (displayed in FITC channel). Full gating strategy for P0 is displayed in Supplementary Fig. 2. Example from cells dissociated from alveolospheres at Passage 0, fibrotic explant. **G** Quantification of proSP-C expression in total living cells from lung organoids in same flow cytometry measurement over the course of 3 passages. Statistical analysis showed neither significant difference in proSP-C expression between P0 and P3 (two-way ANOVA ns, *p* = 0.4915) nor difference between cells derived from fibrotic explants (IPF and secondary fibrosis, turquoise) or healthy tumor-distant lung tissues (red). Cells derived from *N* = 5–7 fibrotic tissues and *N* = 4–6 tumor-distant control tissues. Images were acquired at maximum available magnification without oil immersion due to technical constraints of whole-mount organoid imaging; this may limit spatial resolution of subcellular staining patterns

**Fig. 5:**
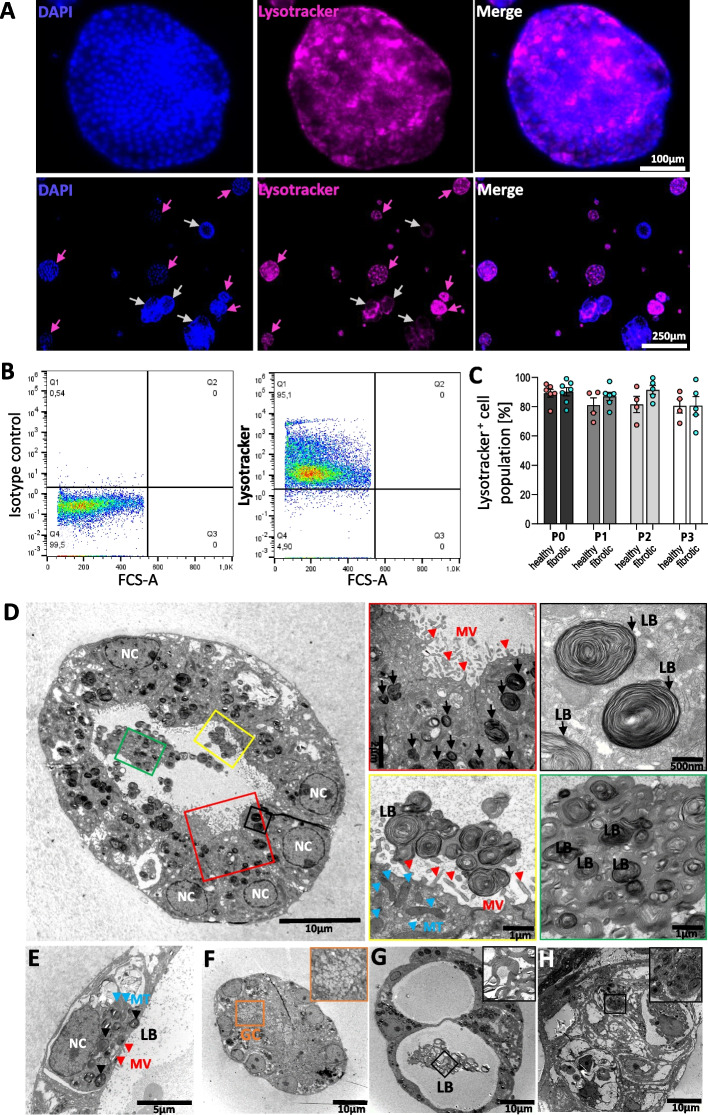
Presence of lamellar bodies in primary human lung organoids indicates phenotype of AT-II cells in alveolospheres. **A** Whole-mount live immunofluorescence staining exhibits the presence of acidic lysosome-related organelles by Lysotracker in cells of the surface layer of AT-II organoids (example from P1, 12 d, fibrotic explant). All replicates (*N* = 5 fibrotic tissues, *N* = 4 healthy controls from tumor-distant tissues, P0-P3) of organoid cultures show signals for intracellular Lysotracker dye in live cell imaging of alveolospheres (upper panel). Lower panel shows differences between highly-positive Lysotracker signals (pink arrows) vs. less pronounced presence of lamellar bodies (grey arrows). Data shown are representative for three independent experiments. Scale bar = 100 µm or 250 µm, respectively. **B** Representative flow cytometry gates for Lysotracker Green DND-26 staining of cell derived from alveolospheres at P2, 10 d from a fibrotic lung explant. **C** Quantification of Lysotracker-positive cell population over different culturing passages. Two-way ANOVA analysis did not demonstrate significant alterations of presumably lamellar body-containing AT-II cells between passages P0 and P3 (ns, *p* = 0.1391). Cells derived from *N* = 5–6 fibrotic tissues and *N* = 4–6 tumor-distant control tissues. **D** Representative transmission electron microscopy (TEM) images of monocystic alveolosphere (small); Overview: scale bar = 10 μm; higher magnifications as indicated. NC = nucleus. Microvilli (MVs, red window and red arrow heads) were observed in spheres at the apical lumen. Mitochondria (MT) displayed with blue arrow heads. A monocystic organoid from fibrotic origin displays accumulations of numerous lamellar bodies on ultrastructural level (LB, black). Black window shows intracellular LBs, while yellow and green window display extracellular LBs. Qualitative analysis displays LBs in all investigated samples (*N* = 4 fibrotic tissues, *N* = 2 healthy controls from tumor-distant tissues, P0-P1) independent of organoid morphology or tissue origin. High magnification images in TEM show single AT-II cell from a monocystic organoid from fibrotic origin. NC (white), MV (red arrow heads), MT (blue arrow heads) and LB (black). Scale bar = 5 μm. **E** Details of a single AT-II cells within a monocystic alveolosphere from fibrotic origin. Scale bar = 5 μm Presence of NC, MTs, LBs and MVs. Structural diversity between AT-II alveolospheres displayed in TEM is further displayed by small lumenless (**F**), small polycystic (**G**) or large honeycomb-structured alveolospheres containing intermediate filaments (**H**); scale bars each 10 μm

All statistical analyses were performed with GraphPad Prism (Versions 8.4.3 and 10.2.3, GraphPad Software, San Diego, USA). Data are presented as mean ± standard deviation (SD) unless stated otherwise. All data were tested for normality prior to statistical analysis by Shapiro–Wilk test. As the datasets did not deviate from normal distribution, parametric statistical tests were applied accordingly. For comparisons of more than two groups with a single factor (e.g. quantification of single- vs. double-positive cytospin fractions in Fig. 1C, basal glycolysis, compensatory glycolysis and ATP production in Fig. 7), a one-way ANOVA was applied followed by Tukey’s multiple-comparison test. For experiments with two independent factors (e.g. passage number and pathological background in colony formation rate, flow-cytometric marker expression across passages, viability and cytotoxicity assays, and Annexin-V staining in Figs. 2, 3, 4 and 5), a two-way ANOVA was used. When appropriate, Sidak’s or Tukey’s post-hoc tests were performed to determine pairwise differences. When datasets contained missing values or unequal sample sizes across repeated measures (e.g. HT-I-56 expression across passages in Supp. Figure 3), a mixed-effects model (restricted maximum likelihood) was employed to account for random effects of patient ID, followed by Tukey’s multiple-comparison test. Paired comparisons within the same donor (e.g. pre- vs. post-cryopreservation viability) were analysed using two-tailed paired Student’s t-tests. Significance was defined as *p* < 0.05. Exact p-values are reported in the figure legends with the following notation: *p* < 0.05 (*), *p* < 0.01 (**), *p* < 0.001 (***), and *p* < 0.0001 (****). All experiments included at least duplicate technical replicates, and no data were excluded.

## Results

### Magnetic bead sorting yields highly pure AT-II and AT-I epithelial cells

From 63 patients (22 controls, 15 IPF, 26 secondary fibrosis), AT-II and AT-I epithelial cells were successfully isolated by MACS against cell-specific plasma membrane markers HT-II-280 or HT-I-56 (Fig. 1A). Immunofluorescence of cytospins directly after magnetic sorting showed 89% HT-II-280^+^ and 91.3% proSP-C^+^ cells with a total of 83.7% double-positive population in HT-II-280-sorted cells on average (Fig. 1B–C). This underlines a significant contrast (****p < 0.0001) to unsorted lung cells (3.1% HT-II-280^+^ and 49.4% proSP-C^+^ cells) displaying a 2.9% double-positive population in total lung cells. Furthermore, also flow cytometry confirmed strong enrichment of HT-II-280^+^ and proSP-C^+^ cells compared to unsorted tissue (Fig. 1D, Supp. Figure 1). In a similar sorting approach, similarly, the isolation of AT-I cells yielded 93.0% HT-I-56^+^ cells (Fig. 1E–F) in contrast to 16.7% HT-I-56^+^ cells in the unsorted lung cell fraction (***p = 0.0079), indicating robust applicability of the MACS strategy for alveolar epithelial subsets. This isolation workflow was robust across > 60 patient samples and can be readily adopted for large-scale tissue cohorts, supporting reproducibility of the enrichment strategy across a larger tissue cohort.

### HT-II-280^+^ cells form 3D alveolospheres with high colony formation efficiency and diverse morphology following culturing optimization

To optimize 3D culturing conditions for primary human HTII-280⁺ alveolar type II (AT-II) cells, we initially adopted the protocol by *Konishi *et al*.* employing a 1:1 ratio of SFFF medium and Matrigel. While this ratio supported initial dome formation, the larger dome volumes (100–130 µl) used in our setup gradually collapsed, causing organoids to sink and contact the rigid plastic surface. Upon contact, cells at the dome–plastic interface lost their cuboidal morphology, detached from the surrounding matrix, and began spreading along the surface. These morphological transformation into a basal-like epithelial phenotype (Fig. 2A) were accompanied by the expression of Cytokeratin 17 (KRT-17) and Cathepsin E (CTSE) while maintaining expression of Epithelial Specific Antigen (ESA)/EpCAM after 7–10 days of culture (Fig. 2C). In parallel, HT-II-280 is lost in these dedifferentiated epithelial cellscompletely (Fig. 2B-C), while majority of cells also display decline in alveolar marker proSP-C expression. Increasing the proportion of Matrigel to a 5:8 (medium:Matrigel) ratio stabilized dome geometry, prevented sinking, and maintained proper embedding of organoids throughout 14-day culture.

Therefore, freshly isolated AT-II cells embedded in Matrigel in a 5:8 (medium:Matrigel) rapidly formed organoids within several days (Fig. 3A). The cultivation of HT-II-280^+^ cells resulted in the formation of both alveolospheres as previously described [27, 29]. Organoids formation reproducibly occurred from both control and fibrotic samples over multiple passages (Fig. 3B-C). The colony formation rate (CFR) was initially lower in controls (41.5%) compared to fibrotic explants (65.4% at P0), but rose significantly in subsequent passages, reaching 79.6% in controls in P1 (****p < 0.0001) and 91.4–94.3% in fibrotic organoids in P1 and P2 (Fig. 3C, P1 and P2: ****p < 0.0001). Fibrotic tissue-derived AT-II cells tended to form alveolospheres more efficiently across passages (P0: ****p < 0.0001; P2: ***p = 0.0002; P3:*p = 0.0373) than cells form tumor-distant “healthy” control tissue. Notably, colony formation efficiency was consistent across independent donors and passages, supporting scalability of the culture workflow for comparative ILD studies.

Interestingly, organoids displayed heterogeneous morphologies, even within same passages and patients. Primary alveolospheres vary both in size, ranging from 50µm to 600 µm, as well in structural morphology: monocystic and polycystic alveolospheres with visible lumina were frequently observed, whereas other organoids developed dense, lumenless spheroid structures as indicated by microcomputed tomography (Fig. 3D–E). H&E staining confirmed alveolosphere-like architecture with epithelial polarity and diverse lumen formation (Fig. 3F). This phenotypic variability was consistent across donors and passages, suggesting inherent heterogeneity of human AT-II progenitor-derived cultures.

### Organoids preserve AT-II identity across passages with limited AT-I differentiation

Despite being physiologically diverse, immunofluorescence of whole mount organoids and tissue sections demonstrated robust expression of HT-II-280 and proSP-C up to passage 2 (Fig. 4A–C, Supp. Figure 2). On a single cell level, flow cytometry revealed that 82% of these cells in organoids express HT-II-280^+^ in the plasma membrane at P0 on average (75.9% [control], 88.5% [fibrotic]). Of note, HT-II-280^+^ expression declined to 65.6% at P3 on average in patient cohort (*p = 0.0423; Fig. 4D-E, Supp. Figure 3). However, this reduction was more pronounced in controls (55.8%) than in fibrotic organoids (75.3%, *p = 0.0305). In stark contrast, proSP-C expression remained on a high level and consistently stable across passages and independent of tissue origin (79% [control] 89% [fibrotic] in P0; 84% [control] 93% [fibrotic] in P1; 83% [control] 88% [fibrotic] in P2; 83% [control] 80% [fibrotic] in P3) (Fig. 4F–G). Hence, we can confirm that the system reliably maintains AT-II identity and can be reproduced across multiple laboratories.

Together, these data indicate preservation of key AT-II–associated marker expression at the population level across passages. A moderate increase in AT-I marker HT-I-56 was detected by P3 (3.7% at P0 vs. 15.1% at P3, *p* = 0.0316; Supp. Figure 4). Dual-positive cells (proSP-C^+^/HT-I-56^+^) appeared in later passages, suggesting limited AT-II–AT-I transition while maintaining overall enrichment of AT-II–associated phenotypes.

### Lamellar bodies confirm AT-II ultrastructural identity

LysoTracker® staining consistently identified lamellar body (LB)-positive cells by histology and flow cytometry across passages, with 89.7% LB^+^ at P0 and 80.5% at P3 independent of tissue origin(Fig. 5A–C). TEM further provided ultrastructural confirmation of AT-II identity, revealing numerous LBs, nuclei, mitochondria, and apical microvilli (Fig. 5D–H) with no quantitative differences between fibrotic and non-fibrotic tissue origin (Supp. Figure 5). Dense-core LB granules were abundant in both control- and fibrosis-derived organoids. LB-like material was also observed in some organoid lumina, reflecting surfactant-related activity. The presence of lamellar bodies across all patient-derived cultures provides a reproducible ultrastructural hallmark of AT-II-associated differentiation states, supporting phenotypic characterization of the epithelial organoids.

### Cryopreservation preserves viability and organoid-forming capacity

**Fig. 6:**
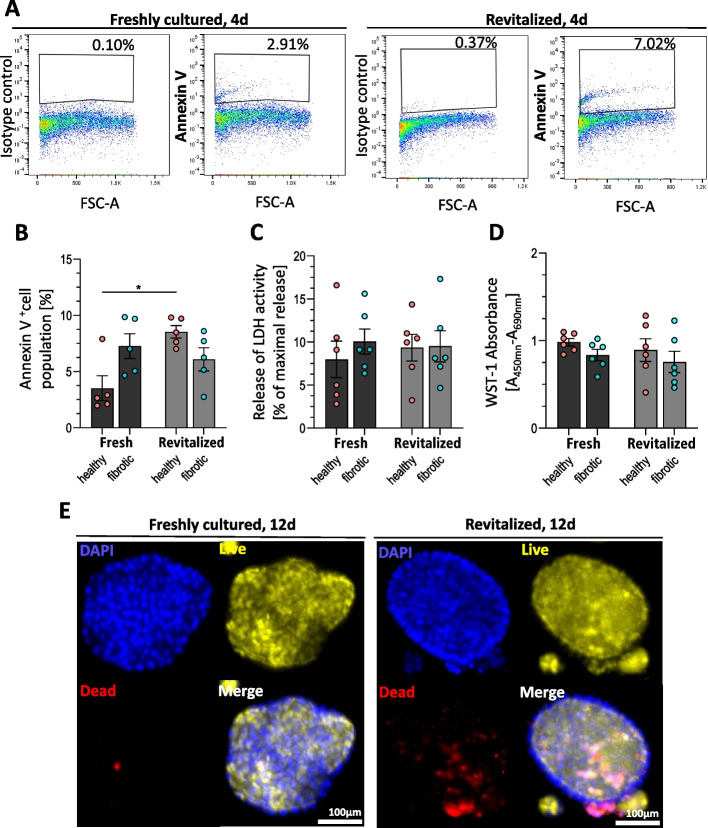
Revitalization and re-culturing of cryopreserved AT-II cells. **A**.Exemplary FACS analysis of dead (Annexin V^+^) AT-II cells derived from alveolospheres prior and post cryopreservation for 28 d at −150 °C. Freshly isolated and cultured AT-II cells from P0 (at 4 d culturing) vs re-cultured cells 4 d following revitalization, P1. **B** Quantification of Annexin V^+^ AT-II cells from FACS analysis. Patient-matched (*N* = 5 fibrotic tissues and *N* = 5 tumor-distant control tissues) comparisons of dead cell percentage before and after cryopreservation (each 4 d in P0 and P1) showed difference as displayed by two-way ANOVA (F(1,8) = 3.9284, *p = 0.0044 for effect of pathological background, F(1,8) = 2.567, *p* = 0.1478 for effect of cryopreservation and F(1,8) = 20.88, p** = 0.0018 for combined effects) and Sidak ‘s multiple comparison test (indicated asterisks). **C** Quantification of cytotoxicity measured by LDH-Assay in cell culture media of matrigel domes containing AT-II cells. Release of LDH activity was measured at 4 d in P0 (freshly cultured) and 4 d in P1 (post cryopreservation). LDH values are represented as the relative amount of released LDH activity compared with the totally lysed control. *N* = 6 fibrotic tissues and *N* = 6 tumor-distant control tissues. Data are expressed as means ± SD. Two-way ANOVA (F(1,10) = 0.04342, *p* = 0.8391 for effect of pathological background, F(1,10) = 0.5485, *p* = 0.4770 for effect of cryopreservation and F(1,10) = 0.4050, *p* = 0.5388 for combined effects) and Sidak ‘s multiple comparison test. Release relative to positive control which is generated by incubating one matrigel dome for 45 min with SFFF medium containing 1% Triton X-100. **D** Quantification of viability before and after cryopreservation by WST-1 assay’s absorbance at 450–690 nm. Measurements at 4 d in P0 (freshly cultured) and 4 d in P1 (post cryopreservation). *N* = 6 fibrotic tissues and *N* = 6 tumor-distant control tissues. Two-way ANOVA (F(1,10) = 0.2.441, *P* = 0.1493 for effect of pathological background, F(1,10) = 1.249, *p* = 0.2899 for effect of cryopreservation and F(1,10) = 0.0021, *p* = 0.9642 for combined effects) and Sidak ‘s multiple comparison test. **E** Representative images of immunofluorescent Live/Dead staining of AT-II organoid surface. Alveolospheres were re-cultured for 12 d following revitalization (P1. Patient-matched alveolospheres from initial culture P0 serve as reference. In total, *N* = 4 fibrotic and *N* = 4 healthy tissue-derived AT-II cells. Nuclei were stained with DAPI

Following cryostorage at − 150 °C for ≥ 28 days, revitalized und re-domed AT-II cells retained high viability and colony-forming potential. Annexin-V staining showed a small increase in apoptotic cells for control-derived organoids (*p = 0.0044) but not for fibrotic samples (*p* = 0.6497; Fig. 6A–B). LDH-assays indicate that cytotoxicity remained unchanged after thawing (Fig. 5C). Moreover, WST-1 assays confirmed preserved metabolic activity (Fig. 6D). Concordantly, Live/Dead staining of alveolospheres formed after revitalization showed minimal cell death based on cell surface evaluation, similar to fresh cultures (Fig. 6E). Together, these findings demonstrate the suitability of cryopreserved AT-II organoids for viability-preserving storage and subsequent re-expansion.

### Fibrotic AT-II organoids undergo glycolytic reprogramming

The increased colony formation efficiency observed in fibrotic AT-II organoids suggested enhanced proliferative activity, which often coincides with metabolic shifts toward glycolysis. In idiopathic pulmonary fibrosis (IPF), alveolar epithelial cells exhibit profound mitochondrial dysfunction and glycolytic reprogramming, which are thought to sustain epithelial activation and maladaptive repair responses [35, 36]. Moreover, glycolysis and lactate signaling have been implicated in driving fibrogenic processes under hypoxic and stress conditions [37], while enhanced glycolytic activity was recently shown to be essential for energy production and regenerative capacity in alveolar stem cells [38]. Given these observations and the reported decline in regenerative potential of AT-II cells with disease progression [39], we sought to determine whether our organoid model reflects disease-associated metabolic alterations by analyzing glycolytic and oxidative metabolism using extracellular flux assays.

**Fig. 7:**
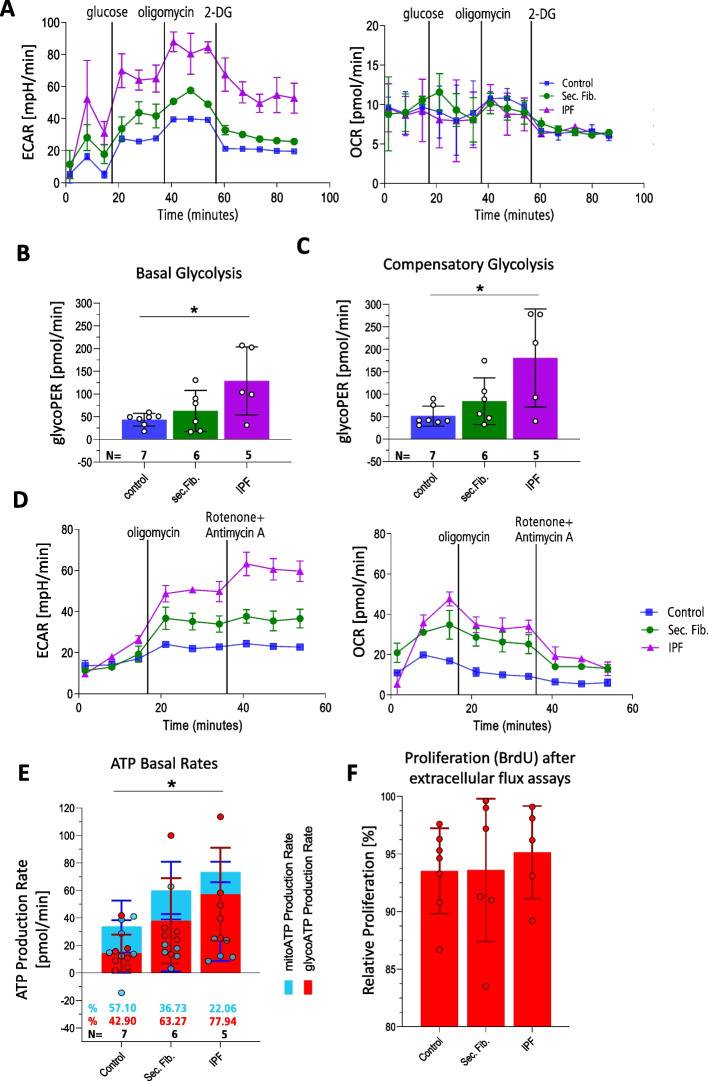
Fibrotic AT-II cells within primary organoids increase glycolysis and decrease level of mitochondrial respiration during end-stage disease. **A** Exemplary measurement of extracellular acidification rate (ECAR) (**A**) and corresponding O_2_ consumption rate (OCR) was measured for primary ATII cells cultured for 12 d. IPF, secondary fibrosis or healthy origin. Data including 18 patients (*N* = 7 tumor-distant control tissues, *N* = 6 s. fibrosis and *N* = 5 IPF tissues) highlights trend towards increased glycolytic activity of IPF-derived AT-II organoids in basal glycolysis rates (**B**) and significant difference in compensatory glycolysis rates (**C**). Data is normalized to control. Basal glycolysis (**B**) assessed by one-way ANOVA (F = 4.954;*p = 0.0233) followed by Tukey’s multiple comparison test (*p = 0.0201 for control vs. IPF; *p* = 0.7577 for control vs. sec. Fibrosis; *p* = 0.0852 for sec. fibrosis vs. IPF). Compensatory glycolysis (**C**) assessed by one-way ANOVA (F = 5.905; *p = 0.0128) followed by Tukey’s multiple comparison test (*, *p* = 0.0109 for control vs. IPF; *p* = 0.6455 for control vs. sec. fibrosis; *p* = 0.0679 for sec. fibrosis vs. IPF). To assess the ATP production rate, measurements were conducted utilizing oligomycin addition to the assay media followed by RotAA addition. Representative ECAR und OCR data (**D**) was utilized to calculate the total ATP production rate (**E**) based on total glyoATP and mitoATP production. The level of calculated proportions of total basal ATP production rates corresponding to glyoATP and mitoATP rate are shown in Fig. E based on same 18 patients. Assessment of ATP production by one-way ANOVA (F = 3.881; *p = 0.401) followed by Tukey’s multiple comparison test (*p = 0.0477 for control vs. IPF; *p* = 0.1228 for control vs. sec. Fibrosis; *p* = 0.4473 for sec. fibrosis vs. IPF). **F** BrdU data shows that proliferation of AT-II cells in organoids is not drastically decreased following Extracellular flux experiments. Relative proliferation [%] displayed, given as (OD_450nm_ after Extracellular flux/OD450_nm_ after Extracellular flux)*100. Data obtained by ELISA as replicates of two; same donors as for extracellular flux measurements

Metabolic analyses were performed using organoids from passages P1 and P2. No passage-dependent differences in basal glycolysis, compensatory glycolysis, or ATP production were observed between P1 and P2, allowing combined analysis of both passages for all subsequent experiments. Indeed, extracellular flux analysis revealed enhanced glycolysis in fibrotic organoids compared to controls. In Detail, extracellular Acidification Rate (ECAR) was consistently higher in IPF- and secondary fibrosis-derived alveolospheres (Fig. 7A–B). Interestingly, glycolysis was significantly (threefold) elevated in AT-II organoids derived from IPF tissue vs healthy control tissue (*p = 0.0201) after glucose injection while same tendency was present in alveolospheres cultured from tissue of secondary fibrosis cases (1.4-fold increase; *p* = 0.7577). Upon oligomycin stimulation, IPF organoids displayed 3.5-fold higher compensatory glycolysis (*p = 0.0109), while secondary fibrosis organoids showed a 1.6-fold increase (*p* = 0.6455) in glycolytic capacity relative to controls (Fig. 7C). OCR remained largely unchanged, suggesting preferential reliance on glycolysis over oxidative phosphorylation.

ATP rate assays further demonstrated that fibrotic organoids generated significantly more ATP, with IPF samples producing 2.2-fold more (*p = 0.0477) and secondary fibrosis samples 1.7-fold more (*p* = 0.1228) ATP than controls (Fig. 7D–E). Notably, the additional ATP was predominantly glycolytic, contributing 77.9% of total ATP in IPF, 63.3% in secondary fibrosis, and 42.9% in control AT-II organoids. Patient-to-patient heterogeneity was evident, as shown in Supplementary Figs. 6 and 7. In addition, we show that cell proliferation (Fig. 7F) remained stable (93–95%) between organoids from “healthy” tissue und IPF-derived tissues) following the extracellular flux assays, indicating comparable cell numbers during extracellular flux measurements.

## Discussion

In this study, we established a defined, cell-line-free, patient-derived 3D lung organoid model generated from primary human alveolar type II (AT-II) epithelial cells isolated from fibrotic and non-fibrotic lung tissues. Using HT-II-280-based magnetic bead sorting, adapted from *Konishi *et al*.* [33], we achieved high-purity AT-II cell populations that were expanded in serum- and feeder-free conditions to form alveolospheres. These organoids displayed key AT-II–associated features—proSP-C production, HT-II-280 localization, lamellar body formation—and maintained AT-II–associated marker expression at the population level across passages.. Compared to existing in vitro systems relying on iPSCs, immortalized lines, or murine cells [26, 28, 40], our approach provides direct access to primary human AT-II cells and preserves patient-specific phenotypic characteristics.

A major strength of our model is the high cellular purity andpreservation of AT-II–associated marker expression over serial passaging.. More than 80% of cells within alveolospheres remained HT-II-280^+^ or proSP-C^+^ across 3D-culture passaging, consistent with human AT-II organoid data [30]. Although HT-II-280–based magnetic sorting provided a highly enriched AT-II population prior to culturing, purity was not absolute. We are aware that using FACS sorting instead of MACS sorting would potentially result in a higher level of purity. However, when using primary human explants, viability is critical for organoid formation. Several studies indicate that AT-II cells are sensitive to shear stress during FACS sorting [4, 33]. As our goal was to maximize viability for large-scale clinical samples, we opted for this approach. Immunofluorescence of cytospins revealed ~ 89% HT-II-280⁺ cells, while flow cytometry directly following sorting also detected strong enrichment of HT-II-280⁺ cells, however indicating that a small fraction of non–AT-II cells remained after isolation. These observations are consistent with the moderate colony formation rate (CFR) observed in the initial passage (P0), whereas both CFR and AT-II marker-specific labeling in flow cytometry notably increased in P1, suggesting preferential expansion of proliferatively competent AT-II–associated subpopulations under SFFF conditions rather than uniform maintenance of the full initial cellular heterogeneity. We also observed that organoids derived from fibrotic AT-II cells consistently formed in higher numbers and reached greater cell yields than those from healthy donors, despite identical seeding densities. This growth advantage aligns with previous reports describing hyperproliferative, stress-activated epithelial states in fibrotic lungs, characterized by elevated glycolytic activity and mitochondrial dysfunction [35]. These biological differences, together with the increased CFR observed in IPF-derived cultures, initially motivated our metabolic profiling experiments. Ultrastructural analysis of organoids revealed abundant lamellar bodies, confirming the presence of AT-II–associated ultrastructural features [16, 17]. Thus, the combination of magnetic enrichment and medium-driven selection in adapted matrigel-culturing likely results in an AT-II–enriched epithelial population rather than a fully homogeneous AT-II compartment. With our chosen ratio of matrigel to SFFF for initial dome pouring, we in addition overcome mechanical influences on epithelial fate: matrix stiffness and mechanical cues are key regulators of alveolar epithelial differentiation in fibrotic lung tissue. It has been shown that increased rigidity of the ECM can induce AT-II cell dedifferentiation and emergence of aberrant basaloid states, as also observed in vivo in fibrotic regions of IPF [6, 8]. Our in vitro findings mirror this behavior, as direct contact of organoids with the rigid plastic surface triggered loss of AT-II morphology and emergence of dedifferentiated epithelial states with partial basaloid features. The 1:1 ratio led to mechanical stress–induced dedifferentiation andbasaloid-like phenotypic changes —an artifact known to occur when domes collapse and organoids contact plastic surfaces [40, 41] — confirmed by precise marker analysis. The cells became KRT17^+^ CTSE^+^ and retained their epithelial (EpCAM^+^) and partially their alveolar (proSP-C^+^) markers, while simultaneously losing HT-II-280. This phenotype likely represents an intermediate or stress-associated epithelial state rather than a fully established basaloid lineage. To overcome this, we adjusted the matrix composition and found that increasing the medium:Matrigel content to a 5:8 ratio, compared to 1:1 ratio utilized by Konishi et al. [33], provided mechanically stable domes and prevented stiffness-induced reprogramming [41, 42] by maintaining a compliant, fully embedded 3D environment. In the context of fibrotic human explants, which exhibit higher proliferative activity and stronger matrix sensitivity than healthy AT-II cells, this modification proved essential for achieving consistent long-term expansion. Our results underscore the profound plasticity of alveolar epithelial cells and provide a mechanism by which mechanical or matrix-related cues can drive cells towards a phenotype characteristic of chronic lung diseases and failed epithelial repair. In addition, immediate post-MACS flow cytometry analysis using the same HT-II-280 antibody demonstrated preserved antigen accessibility after magnetic enrichment, representing a practical technical advance for workflows that combine MACS-based isolation with downstream analytical flow cytometry. Together, these adaptations refine existing AT-II organoid protocols and enable a reproducible, feeder-free culture framework optimized for primary AT-II cells from fibrotic ILD tissue. Furthermore, differentiation into AT-I occurred solely modestly in later passages, with emergence of HT-I-56^+^/proSP-C^+^ intermediates, indicating retained progenitor potential as described in other alveolar regeneration models [43, 44]. Importantly, these results were reproducible across a large number of patient samples, supporting technical robustness of the workflow across heterogeneous donor material.

We also observed morphological heterogeneity of alveolospheres, which could be categorized as lumen-containing (type A) or dense lobular (type B), similar to murine AT-II-derived spheroids [18] and alveolar epithelial progenitor-derived structures [14], highlighting intrinsic phenotypic variability of primary human AT-II–derived organoid cultures.

An important advance is the demonstration that organoids retained structural integrity and viability after cryopreservation. Cryostorage and successful revitalization were reproducible across both fibrotic and control tissues, demonstrating the feasibility of viability-preserving cryostorage and subsequent re-expansion.. Following storage at − 150 °C, we observed no loss of organoid-forming efficiency, viability, or metabolic activity, supporting the feasibility of biobanking. To our knowledge, this is among the first reports of successful cryorecovery of primary human AT-II organoids while preserving key epithelial characteristics. Future studies will also investigate multiple freezing and thawing-cycles to explore impact of multiple cryopreservation. This reproducibility underscores the technical feasibility of standardized cryopreservation workflows for primary human AT-II organoids.

Most notably, we identified a pronounced consistent metabolic shift in fibrotic AT-II organoids—especially those from IPF—towards aerobic glycolysis, indicating that the organoid system reflects disease-associated energetic alterations.. While extracellular flux assays have been used in murine epithelial organoids [45], their application to primary human AT-II organoids from ILD patients is novel. By adapting extracellular flux analysis protocols from intestinal organoid studies [34], we showed that IPF-derived alveolospheres exhibited elevated basal ECAR, enhanced compensatory glycolysis, and increased glycolytic ATP production. These findings are consistent with previously reported metabolic alterations in IPF epithelial cells, including Warburg-like reprogramming [24, 35, 36]. Extracellular flux assays were restricted to P1–P2 organoids, as we did not observe passage-dependent differences in glycolytic activity or ATP production. This approach minimized potential culture-induced artifacts and ensured that metabolic readouts reflected donor-specific rather than passage-related variation. Because this phenotype was consistently detected across independent donors, our study provides a a framework to study metabolic features of patient-derived alveolar epithelium in vitro. In addition to the Seahorse-based measurements, we performed BrdU incorporation assays before and after extracellular flux experiments as a control to verify that metabolic differences were not confounded by altered proliferation or cell number. Future work will complement these analyses with additional metabolic assays, including targeted metabolomics, lactate and glucose utilization assays, and assessment of mitochondrial respiratory chain function using enzyme activity assays or high-resolution respirometry. Such integrated approaches will allow a more comprehensive characterization of the metabolic reprogramming of primary AT-II cells in fibrosis.

The uncoupling of glycolysis from oxidative phosphorylation suggests disease-associated mitochondrial dysfunction and adaptation to high energetic demands of proliferation and ECM remodeling [26, 37]. *Sun *et al. [25]*.* reported that PDK1-driven lactate accumulation suppresses mitochondrial pyruvate dehydrogenase, reinforcing glycolytic flux. Metabolic studies in IPF have also shown decreased late glycolytic intermediates and elevated lactate [37]. Such metabolic rewiring is implicated in epithelial-to-mesenchymal transition (EMT), apoptosis resistance, and profibrotic signaling. The PDK1–HIF-1α axis promotes glycolysis-dependent myofibroblast differentiation under hypoxic/inflammatory stress [38]. Recent organoid-based work by *Choi *et al. [39] further demonstrated decreased regenerative capacity of IPF-derived AT-II cells with disease progression, highlighting the clinical significance of metabolic dysfunction. Of note, metabolic and identity characterization were performed on partially distinct donor subsets and that this may introduce uncertainty regarding the precise differentiation state of the metabolically profiled organoids.

As a novel cellular model, some limitations must be noted. First, the model relies on end-stage explants, limiting insights into early pathogenic events [5]. Second, patient heterogeneity, despite normalization, introduces variability in sorting efficiency and expansion during cultivation. Third, while canonical markers validated AT-II identity, we cannot exclude selection of proliferatively competent AT-II–associated subpopulations during serial passaging, and future studies using single-cell or genome-wide transcriptomic profiling will be required to resolve cellular heterogeneity and molecular state stability across passages [43, 46]

In addition to genomic stability, future work will need to address the cellular heterogeneity and plasticity inherent to primary AT-II isolates. Recent evidence supports that HT-II-280⁺/EpCAM⁺ alveolar progenitors from adult human lung exhibit significant lineage plasticity: in organoid culture, they can generate dual-lineage bronchioalveolar organoids, giving rise to both alveolar and bronchial cell types, depending on culture conditions [47]. These findings reinforce that phenotypic heterogeneity is an intrinsic feature of adult human lung epithelial progenitors in organoid culture, rather than solely reflecting contamination by proximal airway cells. Furthermore, reviews of adult lung-derived organoid systems [48, 49] provide a comprehensive framework for understanding such heterogeneity as an intrinsic feature of human lung epithelial progenitors. Finally, development of fetal human alveolar organoids [49] confirms the technical feasibility and robustness of genuine human alveolar organoids under defined conditions, reinforcing that our adapted protocol is within the currently established range of lung organoid biology. Incorporating stromal or immune components via co-culture or organoid-on-chip approaches could improve physiological relevance and enable modeling of epithelial–mesenchymal and epithelial–immune interactions [31, 50, 51]. Furthermore, possible transdifferentiation of AT-II cells into KRT-17^+^ KRT-5^+^ basal cells under profibrotic mesenchymal or TGF-β signals [32] underscores the need to explore epithelial plasticity in IPF organoids.

The present study was designed to establish and validate a defined and reproducible culture and cryopreservation workflow for primary human AT-II cells derived from fibrotic lung tissue. Accordingly, the focus was on phenotypic characterization based on established protein markers, ultrastructural features, and functional metabolic readouts, rather than on formal benchmarking against existing culture systems. The preservation of AT-II–associated markers at the population level, ultrastructural features, and reproducibility across a large donor cohort provide evidence for technical robustness of the culture system within the scope of the applied characterization methods. Comparative benchmarking across protocols represents a valuable future direction but is not required to demonstrate the validity of the present system. By allowing direct scalable and reproducible comparison of fibrotic versus control AT-II organoids under patient-matched conditions, our model offers a technically robust in vitro framework for mechanistic studies of epithelial dysfunction in ILD.

## Conclusion

Together, our findings define a reproducible, feeder-free 3D organoid culture framework for primary human AT-II cells that enables expansion and phenotypic characterization under defined conditions. These organoids maintained expression of key AT-II–associated markers and ultrastructural features at the population level and remained viable after cryopreservation, supporting viability-preserving storage and subsequent re-expansion. Importantly, fibrotic organoids consistently displayed a relative shift toward glycolytic ATP production, indicating disease-associated metabolic alterations in patient-derived alveolar epithelium in vitro. By combining technical reproducibility, scalability of the culture workflow, and compatibility with cryopreservation, this system provides a standardized in vitro framework for comparative studies of fibrotic versus control AT-II organoids. While future studies incorporating single-cell or genome-wide profiling will be required to further resolve cellular heterogeneity and molecular state stability, the present work establishes a technically robust basis for mechanistic studies of epithelial dysfunction in interstitial lung diseases.

## Supplementary Information

Supplementary Material 1. Supp. Fig. 1. Gating Strategy of HT-II-280^+^ cells following MACS. Flow cytometry gating strategy for freshly MACSed primary AT-II cells in Fig. 1D. Each Isotype control and stainings were gated to exclude debris, focus on single cellsand then gate on FITC^+^ cells. Exemplary display of unsorted fractions, positive fractions labelled for HT-II-280 and positive fractions labelled for proSP-C. Supp. Fig. 2. Controls for HT-II-280 and proSP-C stainings. Corresponding to images from Figure 3B-C, we provide negative controlsfrom organoid cross-sections as well as positive control for HT-II-280and staining on whole lung tissue in IF. Scale bars= 50µm. Supp. Fig. 3. Gating Strategy of HT-II-280^+^ cell 3D organoid culture. Flow cytometry gating strategy for AT-II cells cultured as organoids in matrigel domes corresponding to Fig. 3D and 3F. Gating strategy included exclusion of debris, focus on single cellsand gating on FITC+ cells in isotype control in sample Aor staining in sample Bper measurement. Similar gating was applied for flow cytometry of Lysotracker in Fig. 4B, Annexin V staining in Fig.5A and HT-I-56 staining in Supp. Fig. 3. Supp. Fig. 4. Minor differentiation of AT-II cells to AT-I cells during 3D-culturing in later passages A. Whole mount immunofluorescence staining displays certain HT-I-56 positive cells on the surface of AT-II alveolospheres. Representative examples of N=5 organoid cultures from IPF patients and N=4 tumor-distant tissues from resections. Though majority of surface level cells from the lung organoid display proSP-C-production in P2, single cells display protein expression of the AT-I cell-specific marker. Scale bar=50µm. B. Direct comparison of HT-I-56 expression utilizing immunolabeling of cryosections from the same tissue/patientbetween P0 and P2 highlights certain differentiation of AT-II to AT-I cells in vitro in 3D culture. Data from N=3 IPF and N=1 healthy controls from tumor-distant tissues, P0-P2. Scale bar=50µm. C. Total cells from lung organoids were separated into single cell suspension and labeled with HT-I-56 ms IgG1 and gt anti-mg IgG1Alexa Fluor-488for flow cytometry. Example from the same tissue/patientbetween P0 and P2. Scale bar=50µm. D. Quantification of HT-I-56 expression. Statistical analysis showed significant difference in HT-I-56 expression between P0 and P3 in cells derived from fibrotic explants, but no difference between cells derived from fibrotic explants. Supp. Fig. 5. Ultrastructure comparison of AT-II organoid morphology from fibrotic vs. from pathologically unsuspicious background. Based on data set from TEM, displayed in Fig4D-H, AT-II organoids from IPF origin vs. tumor-distant control tissues are put in direct comparison. Overview of whole single organoids in TEM. Scale bars= 100µm. For both tissue origins, both organoids containing a lumenand lumenless/filled structures display cells with distinct AT-II features. Independent of tissue origin and morphology, AT-II organoids display pronounced presence of lamellar bodieswithout major ultrastructural differences. Supp. Fig. 6. Entire cohort of extracellular flux measurements: Glycolysis stress test. Cohort for Seahorse assays displayed heterogeneity which causes high deviation between patients per group in Fig. 5B and 5C. For illustration, ECAR data, OCR data and corresponding basal glycolysis and compensatory glycolysis is shown for all 18 patients. For more information on tissue background, see Table 1. Cells either derived from P1 or P2 as indicated. Supp. Fig. 7. Entire cohort of extracellular flux measurements: ATP Rate Assay. Cohort for Seahorse assays displayed heterogeneity which causes high deviation between patients per group in Fig. 5E. For illustration, ECAR data, OCR data, and corresponding ATP rates, divided in glycoATP and mitoATP, are shown for all 18 patients. For more information on tissue background, see Supplementary Table 1. Cells either derived from P1 or P2 as indicated.

Supplementary Material 2. Supp. Table 1. Serum-free feeder-free medium. Components for generation of AT-II cell culturing medium

Supplementary Material 3

## Data Availability

The raw data supporting the conclusions of this article will be made available by the authors, without undue reservation.

## Abbreviations

ABCA3: ATP-binding cassette sub-family A member 3
ANOVA: Analysis of variance
AT-I: Alveolar type I cell
AT-II: Alveolar type II cell
ATP: Adenosine triphosphate
CFR: Colony formation rate
CTSE: Cathepsin E
DAPI: 4′,6-Diamidino-2-phenylindole
DMSO: Dimethyl sulfoxide
DZL: Deutsches Zentrum für Lungenforschung (German Center for Lung Research)
ECAR: Extracellular acidification rate
ECM: Extracellular matrix
EGF: Epidermal growth factor
EMT: Epithelial–mesenchymal transition
EpCAM: Epithelial cell adhesion molecule
ERS: European Respiratory Society
ESA: Epithelial Specific Antigen
FACS: Fluorescence-activated cell sorting
FGF-10: Fibroblast growth factor 10
GT: Goat antibody
H&E: Hematoxylin and eosin
HIF-1α: Hypoxia-inducible factor 1-alpha
Hopx: Homeodomain-only protein x
HT-I-56: Human alveolar type I cell marker 56
HT-II-280/HT-II-280: Human alveolar type II cell marker 280
IF: Immunofluorescence
ILD: Interstitial lung disease
IPF: Idiopathic pulmonary fibrosis
iPSC: Induced pluripotent stem cell
KRT-5: Cytokeratin 5
KRT-17: Cytokeratin 17
LB: Lamellar body
LDH: Lactate dehydrogenase
LDHA: Lactate dehydrogenase A
LAMP3: Lysosomal-associated membrane protein 3
MACS: Magnetic-activated cell sorting
MHH: Medizinische Hochschule Hannover
MS: Mouse antibody (context: anti-HT-II-280 ms monoclonal
MT: Mitochondria
MV: Microvilli
NC: Nucleus
NKX2-1/TTF-1: NK2 homeobox 1 / Thyroid transcription factor-1
OCR: Oxygen consumption rate
P: Passage
PDK1: Pyruvate dehydrogenase kinase 1
PKM2: Pyruvate kinase isoform M2
proSP-C: Pro-surfactant protein C
RB: Rabbit antibody (context: anti-proSP-C rb polyclonal)
RotAA: Rotenone & antimycin A
SFTPB: Surfactant protein B
SFTPC/SP-C: Surfactant protein C
TEM: Transmission electron microscopy
TGF-β: Transforming growth factor beta
TTF-1: Thyroid transcription factor-1 (NKX2-1)
WST-1: Water-soluble tetrazolium-1
