# Supplementary material for: Optimized culture of primary human alveolar type II cell–derived 3D organoids from fibrotic lung tissue with phenotypic and metabolic profiling
Source: Respir Res. 2026 Mar 7;27:164. doi: 10.1186/s12931-026-03610-9 (PMC13067506; doi:10.1186/s12931-026-03610-9)

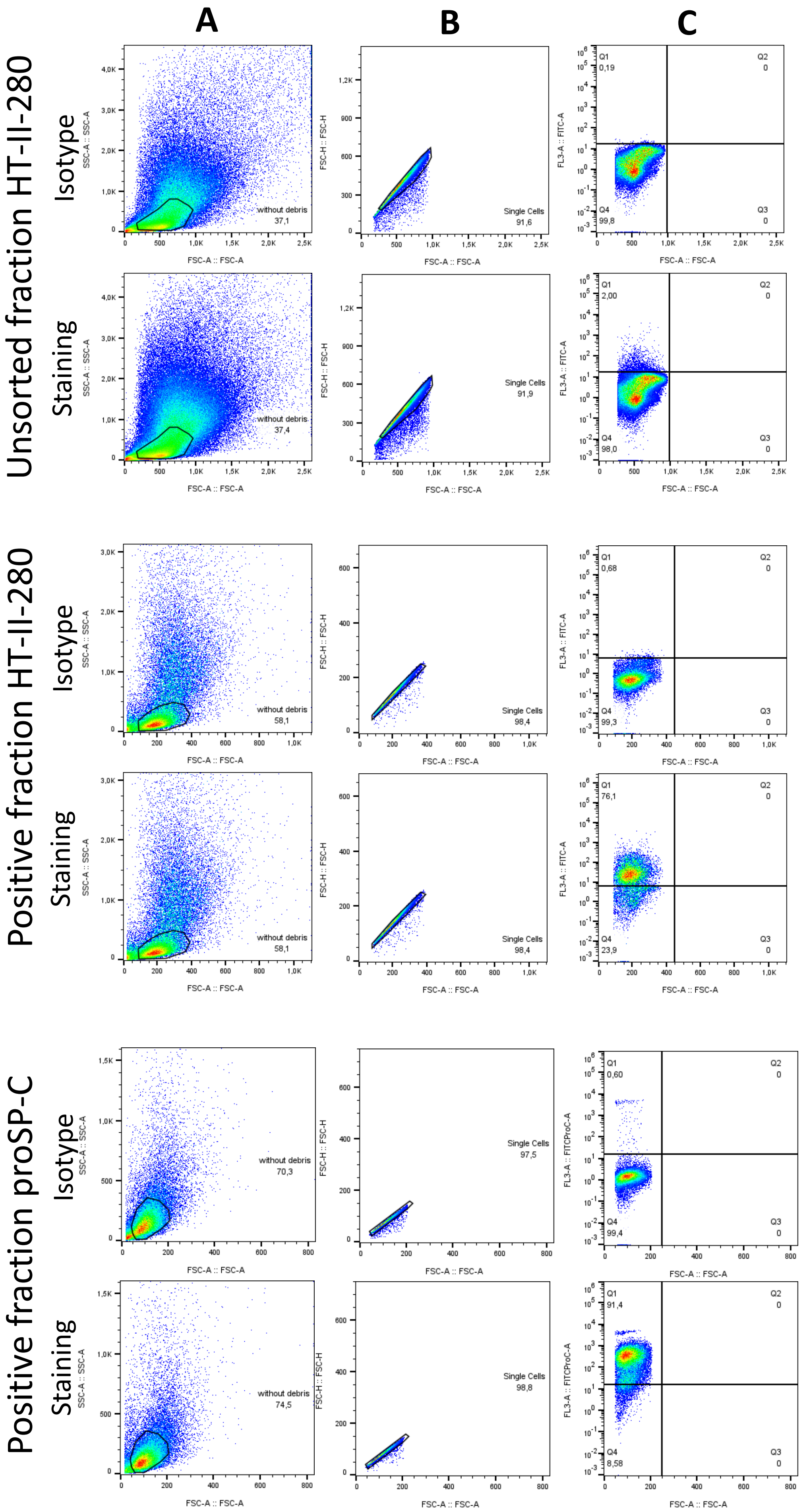
A
B
C
Unsorted fraction HT-II-280
Isotype
Staining
Positive fraction HT-II-280
Isotype
Staining
Positive fraction proSP-C
Isotype
Staining
without debris
Single Cells
Q1
Q2
Q3
Q4
SSC-A :: SSC-A
FSC-A :: FSC-A
FSC-H :: FSC-H
FL3-A :: FITC-A
FL3-A :: FITCProC-A

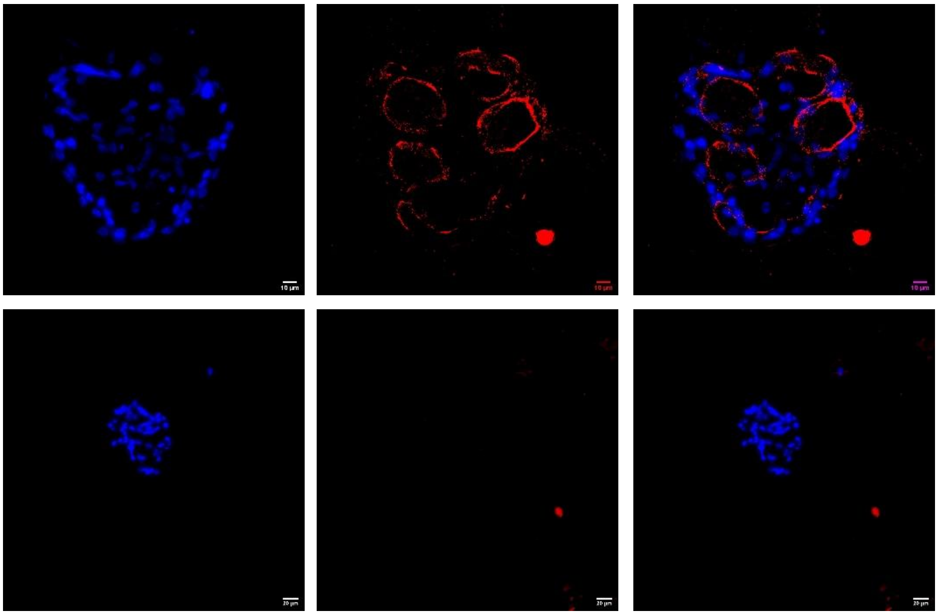

Human lung tissue Positive control (mouse IgM anti-HT-II-280 antibody + secondary AB)

Platzhalter Humanes Gewebe HT-II-280 Färbung

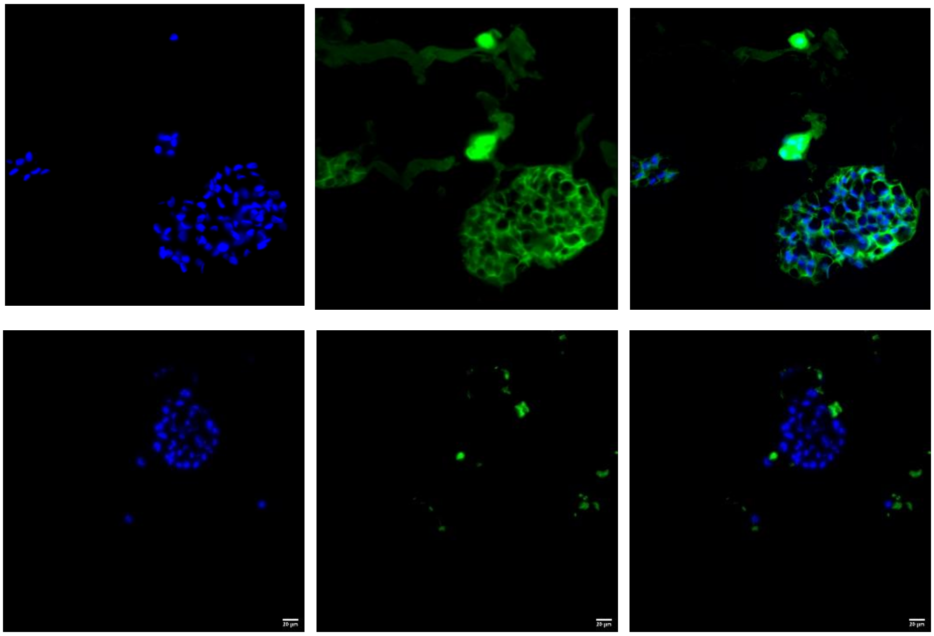

Human lung tissue Positive control (mouse IgM anti-HT-II-280 antibody + secondary AB)

Platzhalter Humanes Gewebe proSP_C Färbung

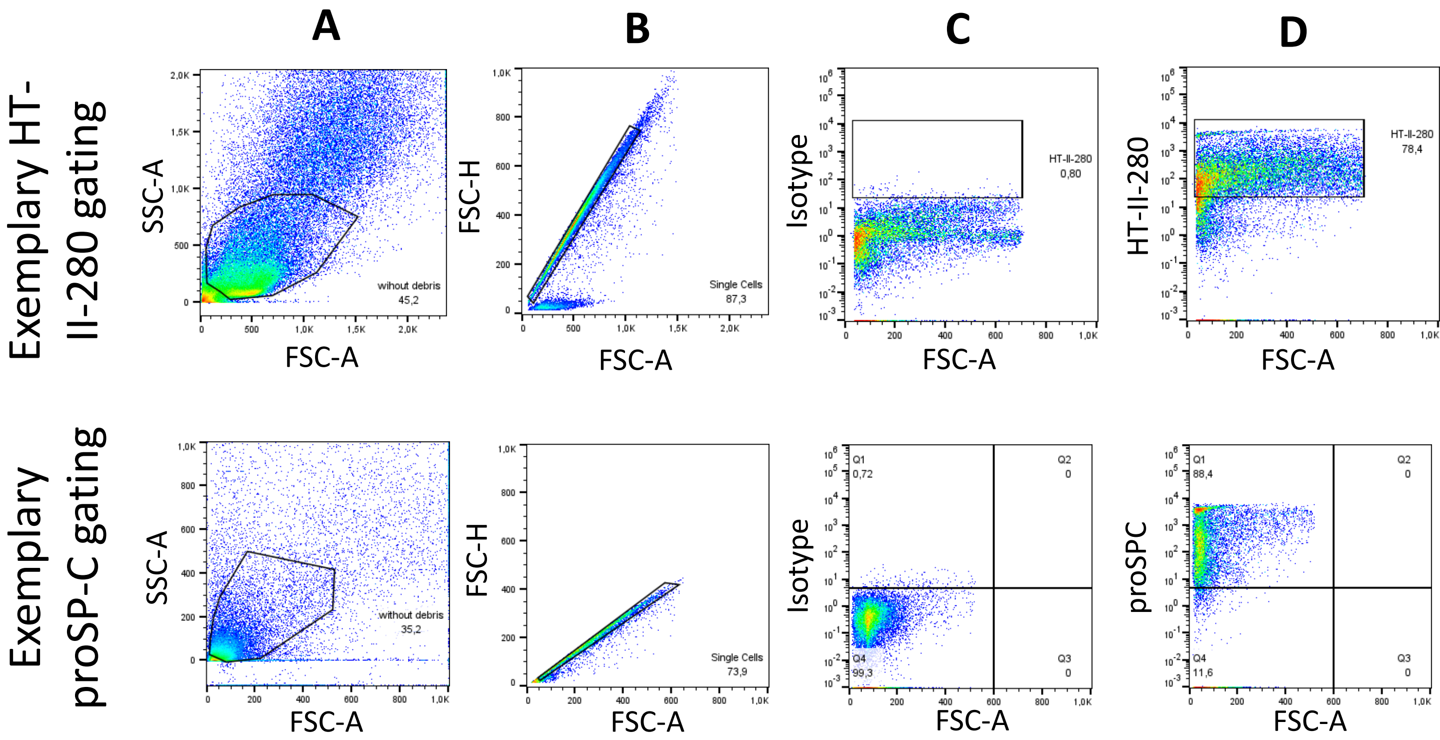
A
B
C
D
Exemplary HT-II-280 gating
Exemplary proSP-C gating
SSC-A
FSC-A
wihout debris
45,2
FSC-H
Single Cells
87,3
Isotype
HT-II-280
0,80
HT-II-280
78,4
without debris
35,2
Single Cells
73,9
Q1
0,72
Q2
0
Q4
99,3
Q3
0
proSPC
Q1
88,4
Q2
0
Q4
11,6
Q3
0

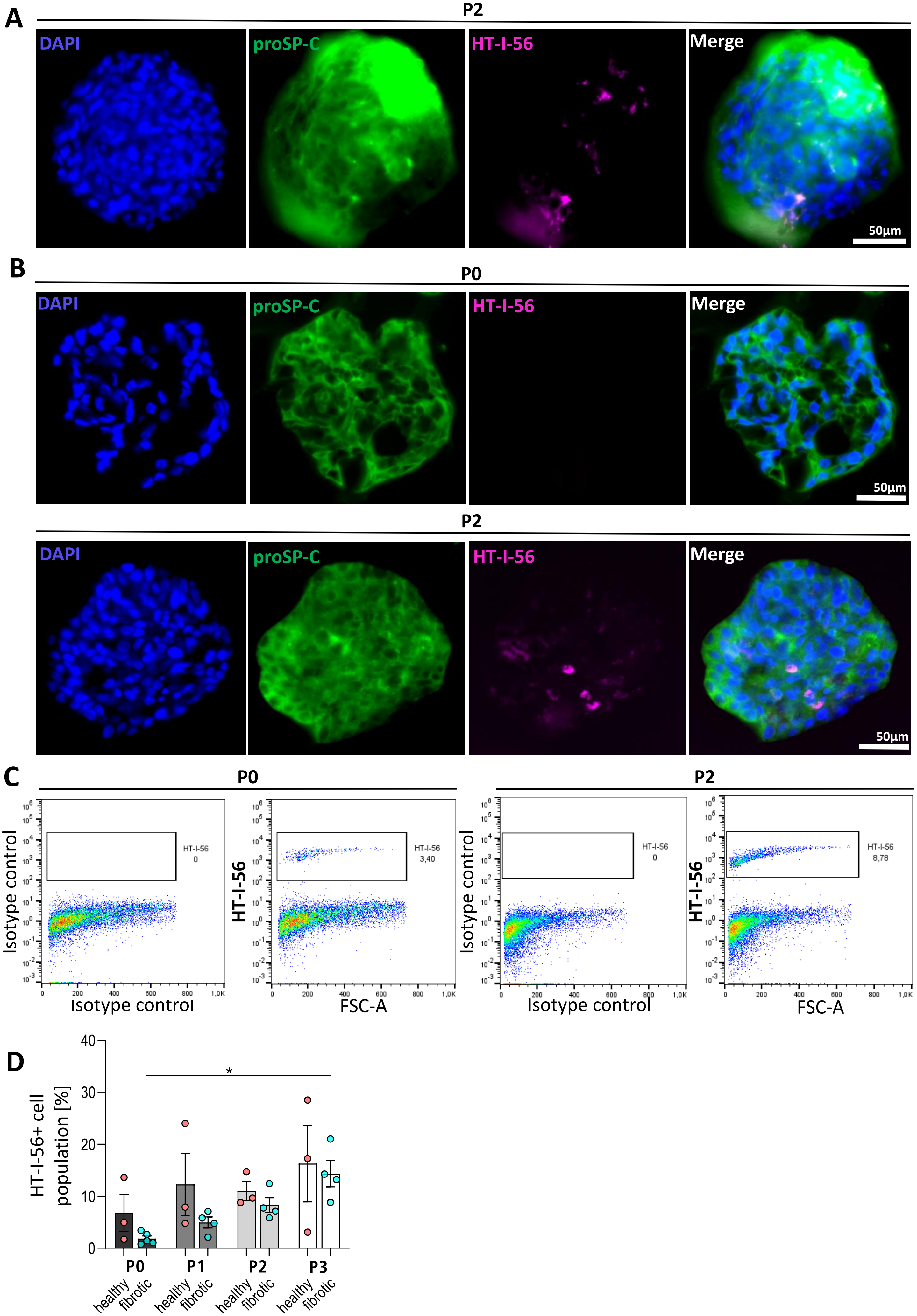

A
P2
DAPI
proSP-C
HT-I-56
Merge
50µm
B
P0
DAPI
proSP-C
HT-I-56
Merge
50µm
P2
DAPI
proSP-C
HT-I-56
Merge
50µm
C
P0
P2
Isotype control
HT-I-56
0
Isotype control
HT-I-56
HT-I-56
3,40
FSC-A
Isotype control
HT-I-56
0
Isotype control
HT-I-56
HT-I-56
8,78
FSC-A
D
HT-I-56+ cell population [%]
*
P0
P1
P2
P3
healthy
fibrotic

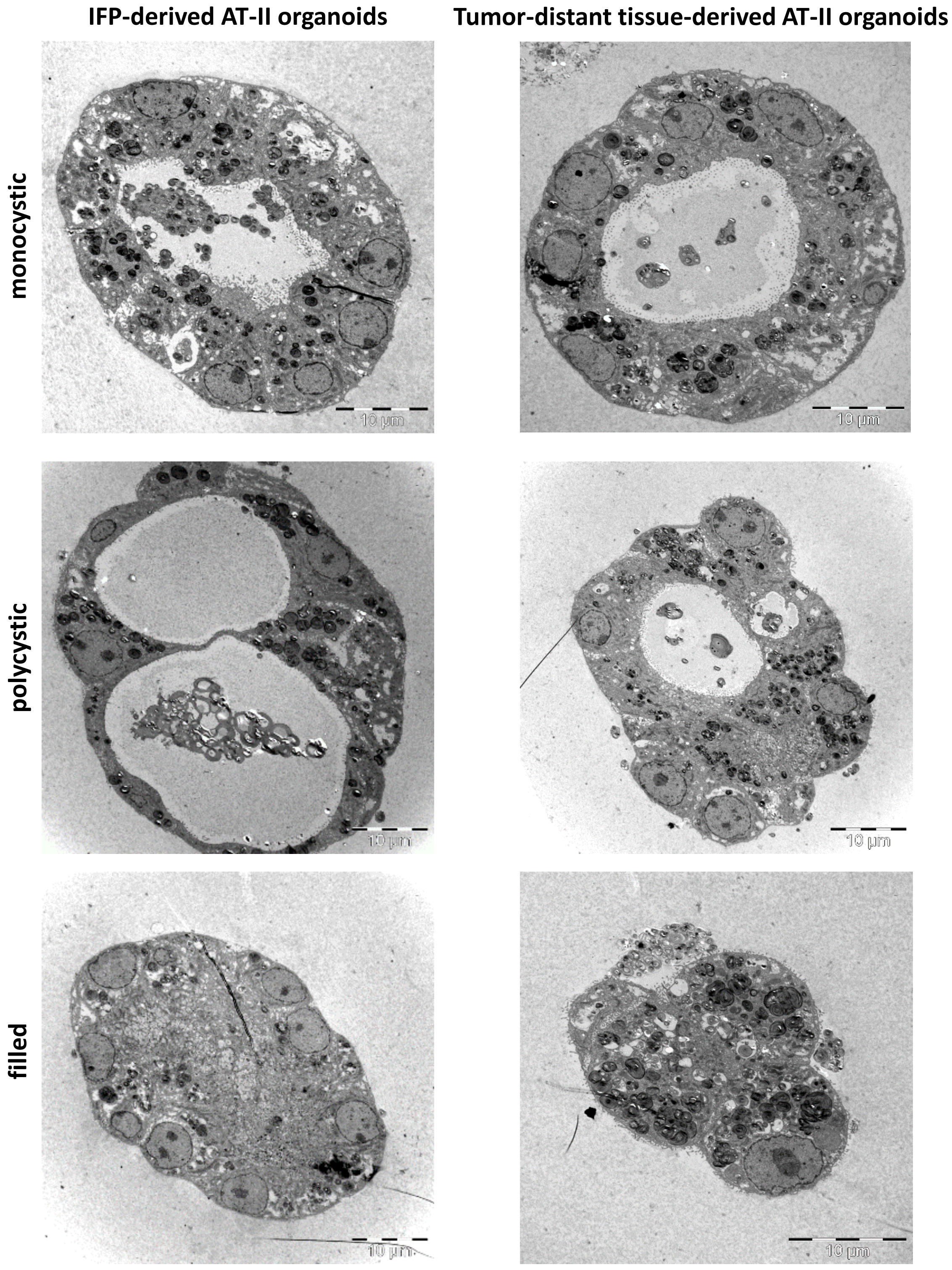
IFP-derived AT-II organoids
Tumor-distant tissue-derived AT-II organoids
monocystic
polycystic
filled
10 µm

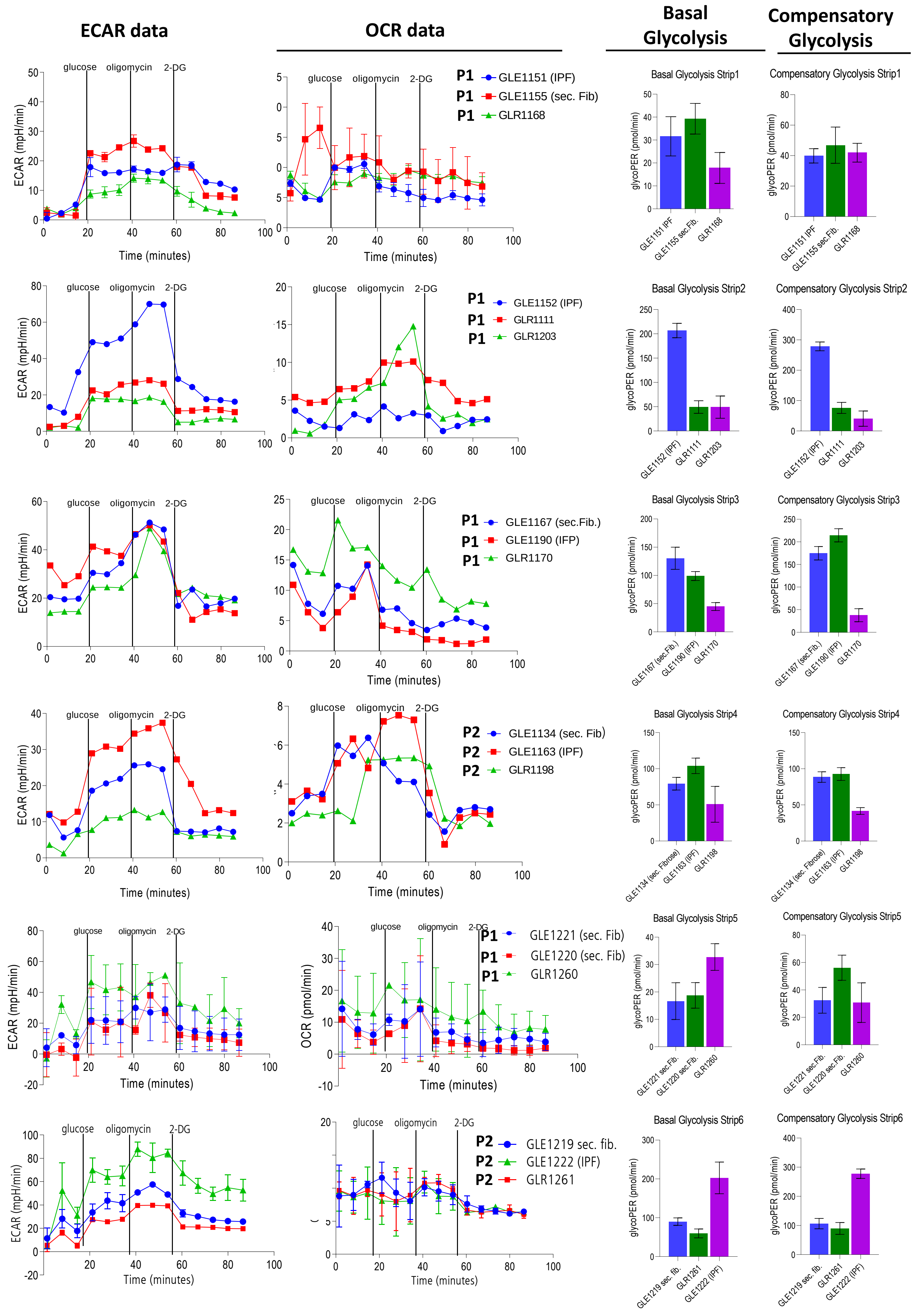
ECAR data
OCR data
Basal Glycolysis
Compensatory Glycolysis
glucose
oligomycin
2-DG
ECAR (mpH/min)
Time (minutes)
OCR (pmol/min)
P1 GLE1151 (IPF)
P1 GLE1155 (sec. Fib)
P1 GLR1168
Basal Glycolysis Strip1
Compensatory Glycolysis Strip1
glycoPER (pmol/min)
GLE1151 IPF
GLE1155 sec.Fib.
GLR1168
P1 GLE1152 (IPF)
P1 GLR1111
P1 GLR1203
Basal Glycolysis Strip2
Compensatory Glycolysis Strip2
GLE1152 (IPF)
GLR1111
GLR1203
P1 GLE1167 (sec.Fib.)
P1 GLE1190 (IFP)
P1 GLR1170
Basal Glycolysis Strip3
Compensatory Glycolysis Strip3
GLE1167 (sec.Fib.)
GLE1190 (IFP)
GLR1170
P2 GLE1134 (sec. Fib)
P2 GLE1163 (IPF)
P2 GLR1198
Basal Glycolysis Strip4
Compensatory Glycolysis Strip4
GLE1134 (sec. Fibrose)
GLE1163 (IPF)
GLR1198
P1 GLE1221 (sec. Fib)
P1 GLE1220 (sec. Fib)
P1 GLR1260
Basal Glycolysis Strip5
Compensatory Glycolysis Strip5
GLE1221 sec.Fib.
GLE1220 sec.Fib.
GLR1260
P2 GLE1219 sec. fib.
P2 GLE1222 (IPF)
P2 GLR1261
Basal Glycolysis Strip6
Compensatory Glycolysis Strip6
GLE1219 sec. fib.
GLR1261
GLE1222 (IPF)

ECAR data **OCR data** **Basal ATP Rates**

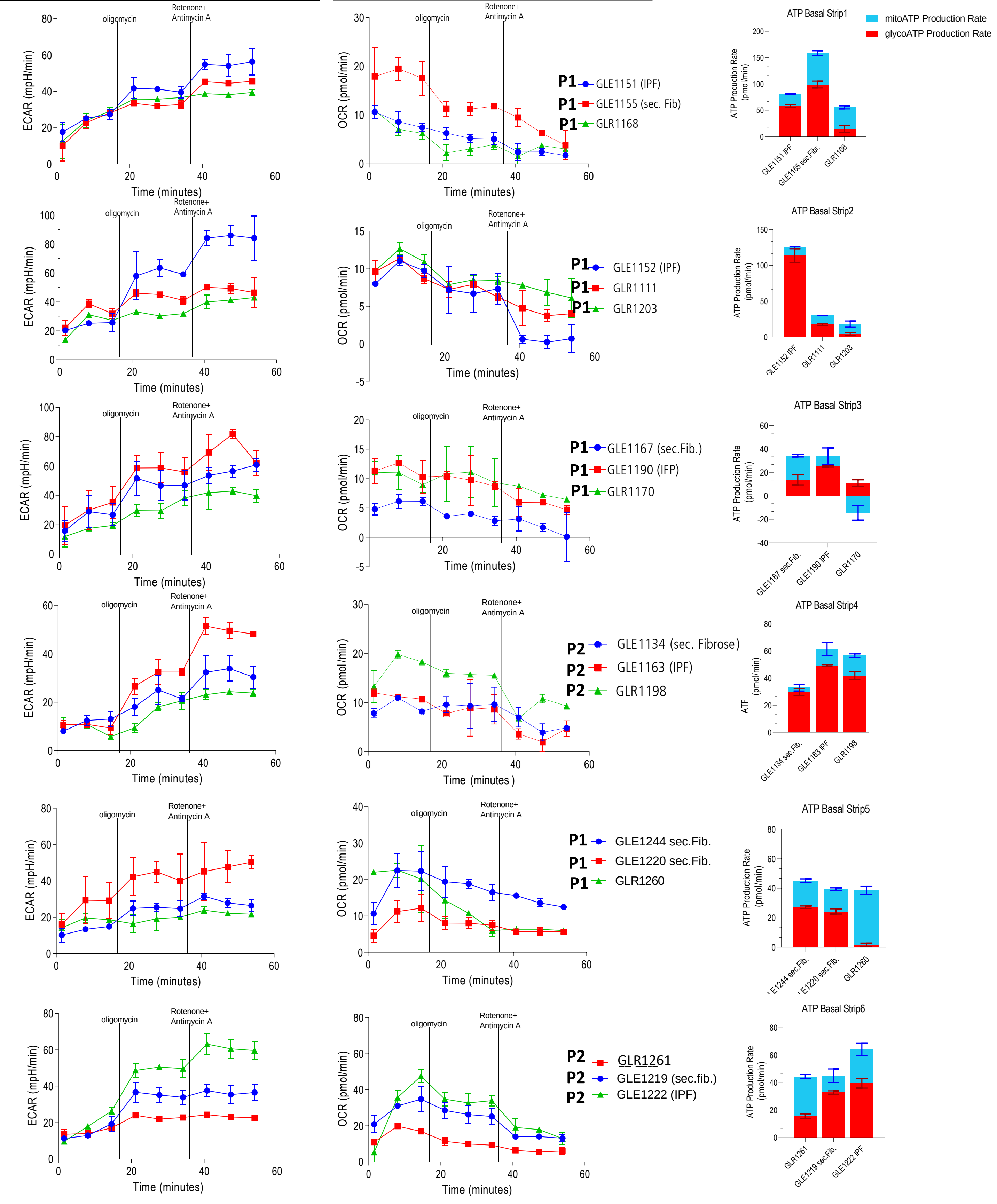

Supplement: Supplementary file 1 — Supplementary Material 1. Supp. Fig. 1. Gating Strategy of HT-II-280+ cells following MACS. Flow cytometry gating strategy for freshly MACSed primary AT-II cells in Fig. 1D. Each Isotype control and stainings were gated to exclude debris, focus on single cellsand then gate on FITC+ cells. Exemplary display of unsorted fractions, positive fractions labelled for HT-II-280 and positive fractions labelled for proSP-C. Supp. Fig. 2. Controls for HT-II-280 and proSP-C stainings. Corresponding to images from Figure 3B-C, we provide negative controlsfrom organoid cross-sections as well as positive control for HT-II-280and staining on whole lung tissue in IF. Scale bars= 50µm. Supp. Fig. 3. Gating Strategy of HT-II-280+ cell 3D organoid culture. Flow cytometry gating strategy for AT-II cells cultured as organoids in matrigel domes corresponding to Fig. 3D and 3F. Gating strategy included exclusion of debris, focus on single cellsand gating on FITC+ cells in isotype control in sample Aor staining in sample Bper measurement. Similar gating was applied for flow cytometry of Lysotracker in Fig. 4B, Annexin V staining in Fig.5A and HT-I-56 staining in Supp. Fig. 3. Supp. Fig. 4. Minor differentiation of AT-II cells to AT-I cells during 3D-culturing in later passages A. Whole mount immunofluorescence staining displays certain HT-I-56 positive cells on the surface of AT-II alveolospheres. Representative examples of N=5 organoid cultures from IPF patients and N=4 tumor-distant tissues from resections. Though majority of surface level cells from the lung organoid display proSP-C-production in P2, single cells display protein expression of the AT-I cell-specific marker. Scale bar=50µm. B. Direct comparison of HT-I-56 expression utilizing immunolabeling of cryosections from the same tissue/patientbetween P0 and P2 highlights certain differentiation of AT-II to AT-I cells in vitro in 3D culture. Data from N=3 IPF and N=1 healthy controls from tumor-distant tissues, P0-P2. Scale bar=50µm [file 12931_2026_3610_MOESM1_ESM.pdf]
