# Supplementary material for: Optimized culture of primary human alveolar type II cell–derived 3D organoids from fibrotic lung tissue with phenotypic and metabolic profiling
Source: Respir Res. 2026 Mar 7;27:164. doi: 10.1186/s12931-026-03610-9 (PMC13067506; doi:10.1186/s12931-026-03610-9)
Supplement: Supplementary file 3 — Supplementary Material 3 [file 12931_2026_3610_MOESM3_ESM.docx]

**1. Colony Formation Rate (CFR) Analysis**

Colony formation rate (CFR) analysis was used to assess the proliferative capacity and viability of lung organoids derived from AT-II cells. During cultivation, representative bright field microscopy images were acquired from five pre-defined fields per sample at each time point, with focus adjusted to the most prominent organoid plane.

Image analysis was performed using the open-source software ImageJ/Fiji (Version 1.54, National Institutes of Health, USA) in a similar approach already utilized by *Chen et al.* (2017)^[S03]^ and *Jain et al*. (2024)^[S04]^ to quantify the CFR of AT-II organoids. Each image was first calibrated by converting pixels to microns based on a known scale bar. A semi-automated procedure was developed to quantify organoid colonies including image pre-processing and segmentation: images were converted to grayscale. To reduce background noise and enhance object contrast, a rolling-ball background subtraction (Process > Subtract Background) with a radius of 50 pixels was applied. Organoids were then segmented from the background by applying the thresholding algorithm "Huang" and converting the image to binary format (Process > Binary > Make Binary). Particle Analysis: colony counting was performed using the "Analyze Particles" function (Analyze > Analyze Particles). A size filter was applied to identify objects corresponding to mature colonies. A colony was defined as a cellular aggregate with a surface area equivalent to a circle with a diameter of at least 50 µm. The minimum particle area was therefore set to 1963 µm (calculated as π⋅(25 µm). To exclude irregular artifacts, a circularity filter was applied (0.40–1.00). The average number of colonies per sample was determined from the five acquired fields. CFR is defined as the percentage of seeded cells giving rise to organoids. The CFR was calculated as the ratio of colonies formed to the total number of cellular structures, expressed as a percentage, CFR(%)=[Number of colonies/Number of total cellular structures per view field]x100. Statistical significance for differences between healthy vs fibrotic cellular origin and differences between passages 0-3 was tested by two-way ANOVA, with p<0.05 considered statistically significant.

## 2. Optimization of Matrigel Dome Composition and Basaloid Marker Validation

For optimization of matrix stiffness, the medium:Matrigel ratio was adjusted from 1:1 to 5:8 (v/v) during dome casting. To induce and validate basaloid-like epithelial outgrowths, freshly isolated primary human AT-II cells were cultured in 1:1 medium:Matrigel on 12 mm glass coverslips (24-well plates) for 7–9 days, with medium changes performed as described above. Partial dome disintegration and epithelial attachment were monitored by brightfield microscopy.

The culture medium, supernatant and Matrigel were carefully removed from the glass coverslips to obtain the accessible, Matrigel-free adherent basaloid-like cell accumulations for subsequent staining. These cells were fixed in 4% PFA (1 h, PBS) and permeabilized/blocked in 1% Triton X-100 and 5% normal goat serum (NGS) in PBS for 3 h at room temperature. AT-II marker loss was assessed using the antibodies described for cytospins and cross-sections as described below. Epithelial identity was confirmed by EpCAM/Epithelial Specific Antigen (ESA) detection using either mouse monoclonal Ber-EP4 (Roche; SN54022, ready-to-use solution) or mouse anti-human NCL-ESA (Leica/Novocastra; 1:100).

Basaloid differentiation was validated by mouse monoclonal KRT17–Alexa Fluor 647 (Santa Cruz Biotechnology; sc-393002 AF647; 1:50; 3 h, RT) and rabbit polyclonal cathepsin E (CTSE; Merck Millipore; HPA012940; 1:100; 3 h, RT), followed by Alexa Fluor 555–conjugated secondary antibody (Invitrogen) and nuclear counterstaining with DAPI (1:100).
Z-stack images were acquired using an APEX100 microscope under identical acquisition settings.

**3.Preparation and immunofluorescent staining of cytospins from freshly MACS-isolated cells**

Following MACS isolation, 30,000-40,000 AT-II or AT-I cells were centrifuged onto Superfrost slides (800 rpm, 10 min, RT) and air-dried for 10 min. Slides were fixed in 4% paraformaldehyde (PFA) for 20 min, permeabilized, and blocked overnight at 4°C in PBS containing 1% Triton X-100 and 5% normal goat serum (NGS).

Preparation of cytospins from freshly MACSed AT-II cells: Fixed and blocked cytospins were incubated overnight at 4°C with primary antibodies: mouse IgM anti-HT-II-280 (1:100, Terrace Biotech, Cat. No. TB-27AHT2-280), rabbit anti-proSP-C (1:500, Abcam, ab271991), or mouse IgG anti-HT-I-56 (1:100, Terrace Biotech, Cat. No. TB-29AHT1-56). After PBS washes, secondary antibodies (1:500; Alexa Fluor® 488 or 555 conjugates, Invitrogen) and DAPI (1:1000, Thermo Fisher) were applied for 8 h at RT. Slides were mounted in Vectashield® mounting medium and imaged using a Keyence BZ-100 fluorescence microscope.

**4. Whole mount immunofluorescence and immunofluorescence of cross-sections labeling of lung organoids**

Organoids cultured for 9–16 days were harvested from Matrigel using Cell Recovery Solution (Corning, Cat. No.: 354253), fixed in 4% PFA, and either processed whole-mount or embedded in OCT medium (Sakura) and cryosectioned at 12 µm thickness.

For both whole-mount and sections, samples were permeabilized and blocked overnight at 4°C in PBS with 1% Triton X-100 and 5% normal goat serum. Optionally, cross-sections underwent antigen retrieval in citrate buffer (15 min, 95°C) as described by *Konishi et al*. ^[S01]^ .Primary antibodies and fluorophore-conjugated secondary antibodies were the same as for cytospins. DAPI was used for nuclear staining and samples were mounted in Vectashield®. Whole organoids were imaged using a Keyence BZ-100 microscope; cross-sections were imaged using an Olympus APX100 system.

As a positive control, human lung tissue was sectioned and stained for HT-II-280 and proSP-C after Cong et al., 2020^[S02]^.

For immunofluorescent labelling of lamellar bodies on the surface on entire organoids, Lysotracker® Green DND-26 (Cell Signaling Technology, Cat. No.:8783S) was directly added to cell culture media for 1 h at 37°C and 5% CO_2._ In addition, DAPI was used for nuclear staining. Whole organoids with matrigel were transferred onto an object slide using a cell scraper and immediately imaged on Keyence BZ-100 microscope.

**5. Flow cytometry analysis of freshly sorted cells or cells derived from AT-II organoids**

Following MACS sorting, single-cell solution was directly utilized for labeling with following cellular fractions described in Fig.2B-D.

*Technical note:* Despite prior magnetic enrichment of HT-II-280⁺ cells, direct flow-cytometric labeling with the same HT-II-280 antibody was feasible. FACS analysis of MACS-sorted fractions consistently yielded 66–88 % HT-II-280⁺ events, indicating that sufficient unoccupied surface epitopes remain accessible for secondary detection. Although partial epitope masking by magnetic beads cannot be fully excluded, careful washing of the MACS product minimizes steric hindrance and enables reproducible downstream analysis.

In addition, single-cell suspensions from mature AT-II organoids were generated as described in *Method section* on 3D organoid culture and at least 30,000 cells were used per sample. All steps were performed at 4°C (unless stated otherwise) and in the dark. Cells were washed three times in MACS buffer (1% BSA, 2 mM EDTA in PBS) by centrifugation (400 g, 5 min) and resuspended in FACS buffer (PBS containing 1% FCS and 1 mM EDTA).

**5.1 General controls and fractions:**

For assessment of cell purity following MACS sorting, three sample types were analyzed by FACS analysis: (i) pre-sorted fraction, (ii) MACS HT-II-280– fraction, and (iii) MACS HT-II-280+ fraction. Unstained controls were included for all fractions. Isotype controls were used for pre-sorted samples (HT-II-280) and for both positive and negative fractions when performing proSP-C intracellular staining.

**5.2 HT-II-280 surface staining**

Cells were incubated with 3 µL mouse IgM anti-HT-II-280 antibody (Terrace Biotech, Cat. No. TB-27AHT2-280; 1:100 in FACS buffer) for 20–30 min at 4°C. After two washes in FACS buffer, cells were stained with Alexa Fluor® 488 AffiniPure Donkey Anti-Mouse IgM (Jackson ImmunoResearch, 1.5 µL/sample, 1:200 in FACS buffer) for 20–30 min at 4°C, washed twice, and resuspended in 300 µL FACS buffer for acquisition.

**5.3 proSP-C intracellular staining**

Cells were fixed in 100 µL Fixation Buffer (BD Biosciences) for 30 min at RT, washed twice with 1× Permeabilization Buffer (diluted from 10× stock with distilled water), and resuspended in 100 µL 1× Permeabilization Buffer containing 5 µL rabbit polyclonal anti-proSP-C antibody (Merck Millipore, AB3786). After 30 min incubation at RT, cells were washed and incubated with 2 µL goat anti-rabbit Alexa Fluor® 488 (Invitrogen, Cat. No. A-11008) in 200 µL 1× Permeabilization Buffer for 30 min at RT. Cells were washed twice in 1× Permeabilization Buffer and resuspended in 300 µL FACS buffer.

**5.4 Lysotracker**® **staining for lamellar bodies**

Cells were directly incubated with 1 µL mouse LysoTracker ® Green DND (Cell Signaling Technology, Cat.No.:8783S-26) for 20–30 min at 4°C, washed twice, and resuspended in 300 µL FACS buffer for acquisition.

**5.5 HT-I-56 surface staining**

Cells were incubated with 3 µL mouse IgG1 anti-HT-I-56 antibody (Terrace Biotech, Cat. No. TB-29AHT1-56; 1:100 in FACS buffer) for 20–30 min at 4°C. After two washes in FACS buffer, cells were stained with goat anti-mouse IgG1 Alexa Fluor® 488 (Invitrogen, Cat. No. A-21121, 1.5 µL/sample, 1:200 in FACS buffer) for 20–30 min at 4°C, washed twice, and resuspended in 300 µL FACS buffer for acquisition.

**5.6 Data acquisition**

Flow cytometry was performed on a MACS Quant10™ cytometer at low flow rate. Alexa Fluor® 488 or LysoTracker ® Green DND was detected in the FITC channel (488 nm excitation, 530/30 nm emission). Detector voltages were set as follows: FSC 320 V, SSC 450 V, FITC (B1) 400 V, FITC secondary detector (B2) 476 V. Data were analyzed using FlowJo™ software (Version 10, BD Biosciences).

**6. Histology and Imaging**

**6.1 H&E staining**

To assess the morphology of cultured alveoleospheres, organoids cultured for 9–16 days were harvested from Matrigel using Cell Recovery Solution (Corning, Cat. FNo.: 354253) and fixed in 4% PFA for 1–2 h at RT. They were firstly embedded in 37°C warm low-melting agarose in plastic chambers. Upon polymerization, agarose gel strips were embedded in paraffin. Sections were made in 2-3 µm thickness, routinely stained for H&E and investigated using brightfield microscopy.

**6.2 Microcomputed tomography** **and Transmission electron microscopy**

Organoids were fixed with 1.5% Glutaraldehyde, 1.5% Paraformaldehyde in 0.15 M HEPES buffer. After treatment with 4% OsO4 and half-saturated uranyl acetate, samples were dehydrated by acetone and finally embedded in epoxy resin according to established methods ^[S0]^. To identify organoids within the resin and to define regions of interest for ultrastructural investigations, embedded samples were scanned by microcomputed tomography at a voxel size of 2 µm (Phoenix NanoTom M, Baker Hughes, Wunsdorf, Germany). Finally, ultrathin sections of a thickness of 80 nm were produced, stained with 3% lead citrate and investigated by transmission electron microscopy (TEM, Morgagni 268, FEI, Eindhoven, The Netherlands).

**7. Viability assays**

Viability, metabolic activity, and apoptosis of AT-II organoid cultures were assessed following reculturing from cryopreservation using four complementary assays: (i) water-soluble tetrazolium (WST)-1 assay to measure metabolic activity, (ii) lactate dehydrogenase (LDH) release assay to determine cytotoxicity, (iii) Annexin-V staining for flow cytometric quantification of apoptotic cells, and (iv) histological Live-Dead-Assay from Invitrogen using whole organoids to assess the number of viable or dead cells on the organoid surface after revitalization from the biobank.

**7.1 LDH cytotoxicity assay**

LDH release was measured using the Cytotoxicity Detection Kit (Roche, Cat. No. 11644793001) according to the manufacturer’s instructions. AT-II organoids were revitalized after one month of cryopreservation, cultured for 4 days, and the culture medium was replaced with fresh medium 24 h prior to the assay. Supernatants were collected and compared to a positive control generated by incubating one Matrigel dome containing AT-II cells for 45 min in medium with 1% Triton X-100. For analysis, 50 µL of culture supernatant from each dome (in duplicate) was transferred to a flat-bottom 96-well plate. The positive control was assayed as undiluted, 1:5, and 1:10 dilutions to ensure absorbance values fell within the optimal range of the plate reader. The LDH working solution was freshly prepared and 50 µL was added to each well. Plates were incubated for exactly 20 min at RT in the dark, and absorbance was read immediately at 492 nm with a reference wavelength of 630 nm. Unconditioned medium with working solution served as a blank control. LDH release was expressed relative to the positive control (adjusted for dilution factors).

**7.2 WST-1 metabolic activity assay**

Metabolic activity was assessed using the WST-1 reagent (Roche), freshly diluted 1:10 in the respective culture medium. Organoids were assayed directly in their culture plate by removing the culture medium and adding 250 µL of WST-1 working solution per well. Plates were incubated for 1 h under standard culture conditions, after which 100 µL of supernatant from each well (in duplicate) was transferred to a new flat-bottom 96-well plate. Working solution without cells served as a blank control. Absorbance was measured at 450 nm (reference: 630 nm).

**7.3 Annexin-V apoptosis assay**

For detection of apoptotic cells, single-cell suspensions were prepared from organoids as described in *method section*. A total of 50,000 cells per sample was stained with Annexin-V conjugated to APC (BioLegend, Cat. No. 640941) according to the manufacturer’s protocol, and analyzed on a MACSQuant® 10 flow cytometer. APC fluorescence was detected at the corresponding wavelength settings, and data were analyzed using FlowJo™ software.

**7.4 Histological Live-Dead Assay**

Live and dead cell populations within AT-II organoid cultures were visualized using the LIVE/DEAD™ Viability/Cytotoxicity Kit (Invitrogen, Cat. No. L3224), which utilizes calcein-AM to detect intracellular esterase activity in viable cells (green fluorescence; Ex 495 nm / Em 515 nm) and ethidium homodimer-1 (EthD-1) to stain nuclei of membrane-compromised cells (red fluorescence; Ex 495 nm / Em 635 nm). Stock solutions were equilibrated to RT prior to use. EthD-1 was diluted to a final concentration of 2 µM by adding 20 µL of 2 mM EthD-1 stock to 20 mL of pre-warmed culture medium, followed by vortexing. Calcein-AM was then added to a final concentration of 1 µM (5 µL of 4 mM stock per 20 mL), vortexed again, and the solution protected from light with aluminum foil. For positive dead-cell controls, organoids in 24-well plates were treated with 500 µL of 70% ethanol for 4 min at RT, followed by a wash with pre-warmed medium. For experimental samples, 150–200 µL of the calcein/EthD-1 working solution was added directly to each well containing Matrigel-embedded organoids. Plates were incubated for 25 min at 37°C and 5% CO₂ in the dark. The staining solution was then removed, and each well was gently washed with 500 µL of pre-warmed medium without penicillin/streptomycin. Organoids were released from the Matrigel by gentle scraping using a sterile cell scraper and transferred onto glass slides. A drop (~20 µL) of PBS was added, and coverslips were applied. To prevent evaporation during imaging, the coverslip edges were sealed with clear nail polish. Samples were immediately imaged using a Keyence BZ-100 fluorescence microscope.

**References**

S01. Konishi S, Tata A, Tata PR. 2022. Defined conditions for long-term expansion of murine and human alveolar epithelial stem cells in three-dimensional cultures. STAR Protocols 3:101447.

S02. Cong X, Nagre N, Herrera J, Pearson AC, Pepper I, Morehouse R, Ji H-L, Jiang D, Hubmayr RD, Zhao X. 2020. TRIM72 promotes alveolar epithelial cell membrane repair and ameliorates lung fibrosis. Respir Res 21:132.

S03. Chen Q, Suresh Kumar V, Finn J, Jiang D, Liang J, Zhao Y-Y, Liu Y. 2017. CD44high alveolar type II cells show stem cell properties during steady-state alveolar homeostasis. Am J Physiol Lung Cell Mol Physiol 313:L41–L51.

S04. Jain KG, Liu Y, Zhao R, Muire PJ, Xi N-M, Ji H-L. 2024. Surfactant Protein-C Regulates Alveolar Type 2 Epithelial Cell Lineages via the CD74 Receptor. J Respir Biol Transl Med 1:10017.

S05. Mühlfeld C, Knudsen L, Ochs M. 2013. Stereology and morphometry of lung tissue. Methods Mol Biol 931:367–390.
